# Examination of Oral Squamous Cell Carcinoma and Precancerous Lesions Using Proximity Extension Assay and Salivary RNA Quantification

**DOI:** 10.3390/biomedicines8120610

**Published:** 2020-12-14

**Authors:** Beáta Scholtz, Doan Vo Minh, Csongor Kiss, Ildikó Tar, Ajneesh Kumar, József Tőzsér, Éva Csősz, Ildikó Márton

**Affiliations:** 1 Genomic Medicine and Bioinformatic Core Facility, Department of Biochemistry and Molecular Biology, Faculty of Medicine, University of Debrecen, 4032 Debrecen, Hungary; scholtz@med.unideb.hu; 2 Biomarker Research Group, Department of Biochemistry and Molecular Biology, Faculty of Medicine, University of Debrecen, 4032 Debrecen, Hungary; doan.vominh@yahoo.com.sg (D.V.M.); kumar.ajneesh@med.unideb.hu (A.K.); 3 Proteomics Core Facility, Department of Biochemistry and Molecular Biology, Faculty of Medicine, University of Debrecen, 4032 Debrecen, Hungary; tozser@med.unideb.hu; 4 Department of Pediatrics, Faculty of Medicine, University of Debrecen, 4032 Debrecen, Hungary; kisscs@med.unideb.hu; 5 Department of Oral Medicine, Faculty of Dentistry, University of Debrecen, 4032 Debrecen, Hungary; tar.ildiko@dental.unideb.hu; 6 Doctoral School of Molecular Cell and Immune Biology, Faculty of Medicine, University of Debrecen, 4032 Debrecen, Hungary; 7 Department of Restorative Dentistry, Faculty of Dentistry, University of Debrecen, 4032 Debrecen, Hungary

**Keywords:** saliva, oral cancer, precancerous lesions, proximity extension assay, mRNA

## Abstract

Saliva is an easy-to access body fluid with high diagnostic potential. The utilization of saliva for oral cancer diagnosis can be an attractive possibility. Besides the oral cancer, it is important to better understand the precancerous lesions such as oral lichen planus (OLP) and leukoplakia (OLK). In order to examine the changes of salivary proteins in controls, patients with oral cancer, and patients with precancerous conditions, proximity extension assay was utilized. Some proteins and functions were characteristic to the examined groups and can serve as a starting point for further biomarker studies. The different nature of OLK and OLP was demonstrated, showing the malignant transformation and the inflammation as the prominent biological processes in the OLK and OLP, respectively. The salivary level of IL6 was verified using quantitative ELISA and the mRNA level was also studied. Elevated IL6 levels could be detected in precancerous groups compared to controls.

## 1. Introduction

Oral squamous cell carcinoma (OSCC) accounts for about 92–95% of all cancer in the oral cavity, 2% of all malignant lesions, and more than 30,000 new cases per year worldwide [1]. OSCC is the cancer of the elderly, the average age at diagnosis being 60 years, with a male:female ratio of 2:1 [2]. Unfortunately, Hungarian men and women exhibit the highest age-standardized rates both for the incidence and the mortality of oral cavity and pharyngeal cancers in Europe, without substantial improvement in the last decades [3]. The development of OSCC takes years, and risk factors such as tobacco usage, cigarette smoking, alcohol, and HPV infection were identified [4,5], with the additional risk factor of betel quid chewing in Asian countries [6]. The genetic factors in the carcinogenesis of OSCC include aberrant overexpression of *NPM*, *CDK1*, *NDRG1*, and *HMGCR*, and suppressed expression of *EF1A*, *NAC*, and *CHES1* [7]. In addition, *TP53* mutations have prognostic value in OSCC, as well as predicting the responsiveness of the disease to platinum-based anticancer drugs [8]. Common locations of OSCC in the oral cavity are the buccal mucosa, the ventral and lateral surfaces of the tongue, the floor of the mouth, and the retromolar region [9]. OSCC can also stem from certain precancerous conditions like oral leukoplakia (OLK) or oral lichen planus (OLP) [1].

Oral leukoplakia (OLK) is defined as tough and adherent white plaques that cannot be scraped off from the oral mucosa. The diagnosis is by exclusion of other diseases that have similar manifestation but no cancer risk, such as oral Candida infections or frictional lesions [10]. Clinically, OLK is divided into 2 main types: homogenous, which are white and flat lesions, and nonhomogenous, which include white spots on a red base, or verrucous and exophytic lesion [11]. Risk factors of OLK include smoking, tobacco chewing, and poor oral hygiene, or it can be idiopathic [11]. Leukoplakia prevalence is 2.6% worldwide and is commonly diagnosed in middle-aged and older men [1]. Whether a leukoplakia lesion will transform into OSCC or not depends on its homogenous or nonhomogenous type, with the homogenous type having a much lower rate of transformation (0.6–5%) compared to 20–25% of the nonhomogenous type [11]. The degree of dysplasia (mild, moderate, and severe) also contributes to whether a leukoplakia lesion will transform into OSCC [11]. Based on global transcriptomics by RNA sequencing, OLK with dysplasia is different from OLK without dysplasia in terms of its molecular pathology: at least 47 genes were found to be differentially expressed between OLK with and without dysplasia, including *SAA1*, *SAA2*, and *KRT31* [12]. Additionally, down-regulation of extracellular matrix (ECM) components is another feature of dysplastic OLK lesion [12].

Oral lichen planus (OLP) is a chronic inflammatory condition of the oral mucous membrane. The etiology of OLP is idiopathic, but it is an immune-mediated disease [13]. According to some reports, OLP occurs after certain viral infections, namely herpes simplex, Epstein–Barr virus, human papillomavirus, and hepatitis C [11]. OLP also shows association with many systemic diseases, such as diabetes mellitus [14], or autoimmune diseases like systemic lupus erythematosus (SLE), rheumatoid arthritis (RA), and multiple sclerosis (MS) [13]. Association with autoimmune diseases might explain in part why elderly women are at higher risk for OLP, which occurs with a female:male ratio of 2:1 [15]. Histologically, OLP is characterized by cytotoxic T cells (CD8+) and helper T cells (CD4+) infiltrating both the epithelium and lamina propria layers. Release of perforin and granzymes by the T cells causes keratinocyte death directly, whereas the release of tumor necrosis factor alpha (TNFα) and interferon gamma induces apoptosis of the keratinocytes [13]. Other cell types involved in the pathogenesis of OLP include dendritic cells, mast cells, and macrophages [16]. Mast cell numbers significantly increase in the lamina propria and at any basement membrane rupture sites in OLP lesions [16]. Dendritic cells are responsible for antigen presentation, while macrophages produce proinflammatory cytokines like TNFα and IL-1β, which contribute to the inflammatory state of OLP [16]. In terms of molecular pathology of OLP, Regulated on Activation Normal T-cell Expressed and Secreted (RANTES) is one of the most widely studied chemokines [16]. RANTES has been shown to be up-regulated in OLP lesion, as a consequence of TNFα released by mast cell degranulation [17]. Besides OLP, RANTES is also involved in the pathogenesis of autoimmune diseases like rheumatoid arthritis or multiple sclerosis [16]. RANTES released by T-cells induce mast cell degranulation in vitro, and the chymase and TNFα from the mast cells can lead to more RANTES release by T cells [17]. This self-sustaining feature of immune activation may account for the cyclical nature of the disease course in OLP [16]. The imbalance between matrix metalloproteases (MMPs) and tissue inhibitors of matrix metalloproteases (TIMPs) is also detected in OLP, setting the background for neoplastic transformation of OLP lesions. [16].

Both OLK and OLP are considered low risk, but potentially malignant conditions, and under certain circumstances both may turn into OSCC [1]. The most important factor in effective treatment of OSCC is to recognize the disease at the early stage. The 5-year disease specific survival (DSS) of patients diagnosed at stage I and II is 92.8% and 79.6%, respectively. If diagnosed at stage III or IV, the DSS become 67.3% and 54.3%, respectively [18]. A problem for the detection of early OSCC lesions is that they present as painless red, speckled, or white patches, which makes it very difficult to differentiate them from other, less dangerous inflammatory conditions in the oral cavity. As a result, 60% of patients with OSCC are diagnosed at stage III and IV, which limits the 5-year survival rate to 10–50% [19].

Saliva, a continuously secreted body fluid that can be obtained noninvasively, is considered to be a rich source of potential biomarkers for various conditions. More than 500 salivary biomarkers have been investigated to date for oral and systemic diseases [20,21]. Identification of salivary biomarkers unique to OSCC has long been the goal of research aimed at early cancer diagnosis, since they would be critical for the effective management of this deadly condition [22,23]. Biomarkers specific for OLK and OLP may also have an important role as early warning signs, especially if correlated with the severity and the capacity for malignant transformation of these conditions [24,25].

## 2. Materials and Methods

### 2.1. Subjects and Salivary Sample Collection

The sample collection was done in accordance with the Declaration of Helsinki and was approved by the Ethical Committee of the University of Debrecen (approval number: 45038/2012/EKU; date: 25 November 2012) and all subjects gave informed consent. Examination of the oral cavity was performed by a licensed dentist, and OLK or OLP were diagnosed according to the 2005 World Health Organization Workshop [26]. The collection of the unstimulated saliva samples for protein quantification was done as described elsewhere [27]. Briefly, donors were asked to avoid eating, drinking, smoking, or using oral hygiene products for at least 1 h before sample collection. The 5 mL of sample collected in 50 mL clean tubes was filtered using a PVDF membrane-containing filter unit (5 µm pore size, Millipore) and 500 µL aliquots of the filtered saliva were stored at −70 °C until further processing. The samples were frozen within less than 30 min after collection. Collection and processing of the unstimulated saliva samples for RNA quantification was done as described before [27]. Control patients for the ELISA and RNA-based studies were the same as before [28]. The age, sex, smoking, and alcohol consumption habits of the recruited patients with OLK or OLP are summarized in Table 1. The type of analyses performed on each sample is summarized in supplementary Table S1.

### 2.2. Relative Quantification of 186 Proteins in Saliva by Proximity Extension Assay

Proteins in the Oncology and Inflammation panels were analyzed by the analysis service of Olink Proteomics (Uppsala, Sweden). Saliva samples originating from 4 groups of 3 donors per group were analyzed. Of each saliva sample, 4 µL was shipped on dry ice to Olink Proteomics, and 1 µL of undiluted and 10-fold diluted saliva, respectively, was used for the analyses. The relative protein quantification was performed using proximity extension assay-based panels. Each panel was able to analyze 92 selected proteins simultaneously, as listed in supplementary Table S2. Using proper internal and external controls, the raw values were normalized for variation between and within runs and were converted into Normalized Protein Expression Units (NPX). These are arbitrary units on log2 scale, allowing the relative quantification of proteins [29]. Proteins which were not detectable in at least 20% of the donors were removed from the analysis, resulting in 146 quantifiable proteins out of the original 184.

### 2.3. Statistical Analysis

Nonparametric Mann–Whitney U test was applied to compare the mean NPX values of the four groups using SPSS 25.0 software (IBM Inc., Armonk, NY, USA). The mean, standard deviation and standard error of mean of the NPX of each proteins were calculated for each studied group. In addition, Levene’s Test for Equality of Variances, *t*-test for equality of mean and 95% confidence interval of the difference were also carries out for each protein. Multiple testing correction in GraphPad Prism (version 7.0b for Mac, GraphPad Software, San Diego, CA, USA, www.graphpad.com) for the False Discovery Rate (FDR), using the two-step method of Benjamini, Kruegel, and Yekuteli on prefiltered protein lists (*p* ≤ 0.05) identified proteins with significantly different expression (FDR ≤ 0.05). For the visualization of the results hierarchical clustering was done and the results were plotted as a heatmap with the help of TBtools [30].

### 2.4. Network Analysis

The network of differentially expressed proteins was generated using String [31]. Default settings were applied and the medium confidence interactions in between the examined proteins were studied. The enriched GO functions and KEGG pathways provided by String were further examined. The gene interaction networks were generated with CluePedia v1.5.7 [32].

### 2.5. ELISA Analysis

Saliva samples originating from 12 patients with OLK and 23 patients with OLP were analyzed. The IL6 protein concentration of saliva samples was analyzed in duplicate by sandwich enzyme-linked immune-sorbent assay using Human IL6 ELISA Kit (Catalog No: 3010018, BioAim Scientific, Markham, ON, Canada), according to the manufacturer’s protocol. Absorbance was measured at 450 nm and concentrations were calculated based on the recorded 7-point calibration curve.

### 2.6. Quantification of Salivary IL6 mRNA by Real-Time Quantitative PCR (qPCR)

Saliva samples originating from 12 patients with OLK and 23 patients with OLP were analyzed. Reverse transcription of salivary RNA and *IL6* qPCR was done as described before, and we used data for the controls from the previous study [28]. Raw data analysis was performed using the ExpressionSuite Software (version 1.0.3 for Microsoft^®^ Windows^®^, Thermo Fisher Scientific, Waltham, MA, USA). Samples with failed amplification for reference genes (as well for *IL6*) were considered to have technical problems, and were omitted from the analysis (4 OLK/OLP samples). Reliably quantifiable samples gave signals in both qPCR replicates for the reference genes and for *IL6*, with low standard deviation (coefficient of variation < 1%) and the replicates both had Ct values within the linear quantification range (Ct ≤ 38). Samples with reliable quantification for the reference genes, but with both *IL6* replicates having Ct values outside the linear quantification range (38 < Ct ≤ 40), or with high standard deviation of replicates, or with only one replicate giving a signal in the qPCR assay, were categorized as borderline, non-quantifiable *IL6*-positive samples. Ct values of these samples were set uniformly to 39 for the calculations and visualization. Samples with reliable quantification for the reference genes, but with no detectable amplification for *IL6* were categorized as *IL6*-negative samples. Normalized *IL6* expression of these samples were set to 0 in the graphs, indicating the limit of detection (LOD). Definition of the quantifiable, positive, and negative samples followed the guidelines for qPCR data interpretation of minimal residual disease quantification for acute lymphoblastoid leukemia, as suggested by van der Velden et al. [33].

## 3. Results and Discussion

### 3.1. Saliva Analysis by Proximity Extension Assay (PEA)

Saliva samples were analyzed by PEA performed at the Olink Proteomics (Uppsala, Sweden) using Oncology II and Inflammation panels. Each of the panels included 92 proteins and 4 internal controls, providing information about 184 proteins altogether (Table S2). The BDNF protein, part of the Inflammation panel, could not be analyzed due to technical issues identified with the BDNF assay, resulting in information about 183 proteins. Our data shows that 7 out of the analyzed 183 proteins does not appear in saliva: interleukin receptors IL2RB, IL15RA, IL22RA1, the interleukins IL2, IL13, and IL33, and the neurturin from the Inflammation panel. Based on the rigorous quality control (QC) [34], all samples passed the QC in both panels (Table S3).

In case of Oncology II panel, 72% of the proteins (66 out of 92) could be detected in more than 75% of the samples, while for the Inflammation panel this value was 45% (41 proteins out of 92) (Table S3). According to Olink Proteomics, in EDTA plasma the detectability is usually more than 90% for proteins in the Oncology II panel, and > 75% for proteins in the Inflammation panel. In our saliva samples, the rate of detectability was lower for both panels, but they were in the same range with the results observed by diPietro et al. using PEA [35]. Our data further supported the observation that the PEA can be the method of choice when comprehensive saliva analysis is required.

### 3.2. Examination of Protein-Level Changes in Saliva Originating from Patients with Oral Cancer or Different Forms of Precancerous Lesions

**Figure 1:**
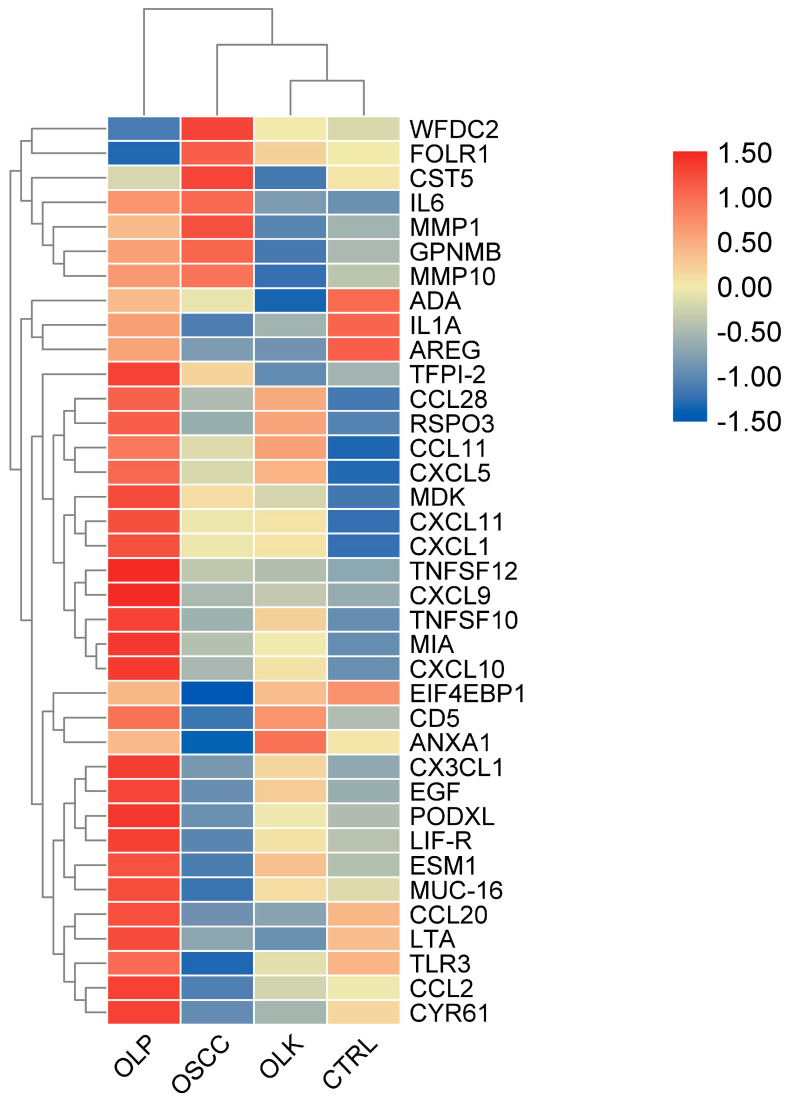
Heat map and hierarchical clustering of proteins, with significantly different expression between two groups of patients, as measured by the proximity extension assay (PEA) method. The proteins are labeled according to their gene symbols.

The level of the proteins quantified in more than 80% of the samples was examined. Out of 146 proteins, 37 showed significant difference between at least 2 studied groups (Figure 1 and Figure S1).

Between the OLP and control group, the differentially expressed proteins were CCL28, CCL11, CXCL5, CXCL11, CX3CL1, PODXL, CXCL10, TWEAK, MCP1 (CCL2), MIA, MK (MDK), RSPO3, TFPI2, and TNFSF10. It was apparent that most of these are chemokines, which correlated with the chronic inflammatory nature of OLP and might indicate the chemotaxis required for cellular infiltration observed in OLP lesions in the histological analyses [13].

Between the OSCC and control group, the following proteins were differentially expressed: ADA, AREG, CCL11, CXCL11, GPNMB, IL1α, IL6, MMP1, MMP10, and TLR3. The presence of IL1α and IL6 was in accordance with the results of our previous study [23]. When the biological functions of the differentially expressed proteins were analyzed further, we found that GPNMB, a glycoprotein overexpressed in OSCC vs. control, is also overexpressed in other types of cancer, such as brain and breast cancer [36]. Matrix metalloproteases are responsible for the degradation of collagen and ECM disassembly, breaking down the basement membrane, which allows cancerous squamous epithelial cells to migrate into deeper tissue layers [37]. Between OLK and the control group, only 2 differentially expressed proteins were observed: CXCL11 and CCL11. Both of them are chemokines involved in many inflammatory and chemotactic processes, being linked to cancer formation [38]. It should be noted that CXCL11 and CCL11 were overexpressed in all disease states compared to the controls.

Protein expression of the different disease states was also compared to each other in order to characterize the progression from one condition to another. Between OSCC and OLP, the differentially expressed proteins were CA125 (MUC16), CCL20, CD5, CX3CL1, CXCL1, CXCL9, CXCL10, CXCL11, CYR61 (CCN1-CCNA2), EGF, ESM1 MCP1 (CCL2), LIFR, PODXL, RSPO3, SLC4A1, TLR3, TWEAK, and WFDC2. The results show a strong signature for chemokines, a common feature of many cancer types where chronic inflammation contributes to the neoplastic transformation [39].

Between OLK and OSCC, the differentially expressed proteins were ANXA1, CST5, EIF4EBP1, GPNMB, IL6, MMP1, MMP10, and WFDC2. WFDC2 was shown to be responsible for epithelial-to-mesenchymal transformation (EMT) and is highly expressed in many tumor cells lines (e.g., ovarian, colon, breast, lung, renal), showing similar expression pattern to GPNMB [40]. The same can be said for ANXA1, which was proven to be involved in OSCC formation [41]. Cystatin 5 (CST5) is a protein expressed in the submandibular and sublingual glands, but not in the parotid gland, and is considered to be an inflammatory marker [42,43].

Between OLK and OLP, only 4 proteins were differentially expressed, namely CYR61 (CCN1-CCNA2), MCP1 (CCL2), MIA, and MK (MDK).

The heat map of Figure 1 indicates the differences among the disease groups suggesting the different nature of OLK and OLP.

### 3.3. Network Analysis of Proteins with Statistically Significant Changes among the Disease Groups

**Figure 2:**
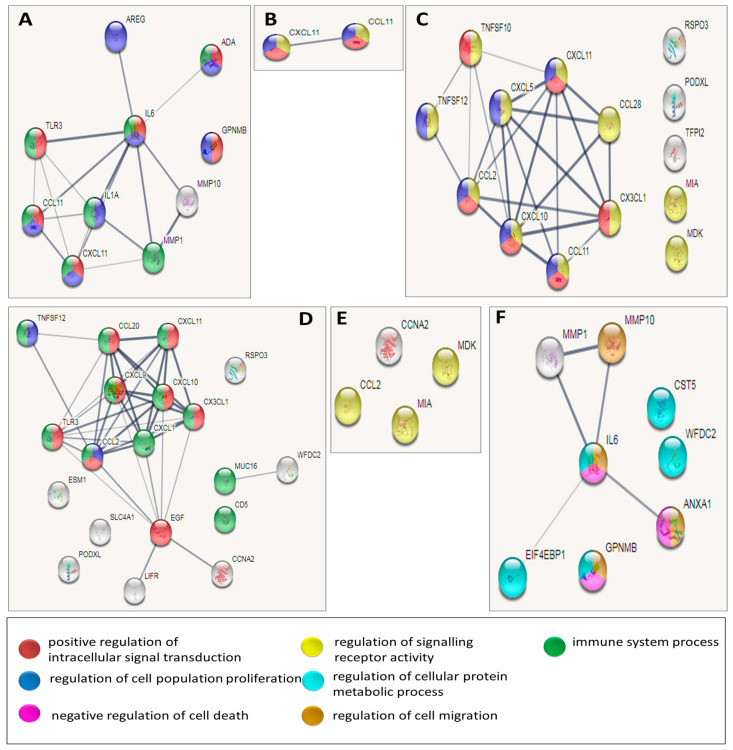
Protein–protein interaction (PPI) network of proteins, with significantly different expression between the patient groups. Each panel shows a network of protein–protein interactions generated by String. Within the network each circle represents a protein, and the lines between the circles represent protein–protein interactions. The line width is correlated with the strength of the supporting evidence for the interaction; the wider the line, the more experimental evidence supports the interaction. The circle color indicates enriched GO function for the protein, explained in the bottom panel. (**A**) Oral squamous cell carcinoma (OSCC) vs. control. (**B**) Oral leukoplakia (OLK) vs. control. (**C**) Oral lichen planus (OLP) vs. control. (**D**) OSCC vs. OLP. (**E**) OLK vs. OLP. (**F**) OLK vs. OSCC.

In order to better understand the biological picture characteristic to OSCC and precancerous conditions, the proteins with statistically significant changes between two groups were used for the generation of protein–protein interaction networks. The String [31] was used for network generation and for the examination of enriched GO functions and KEGG pathways in the networks (Figure 2 and Figure S2).

The differentially expressed proteins between the OSCC and the control groups (Figure 2A and Figure S2) were mostly involved in biological functions that were well-known to be associated with carcinogenesis, such as inflammatory response, regulation of growth factor production, collagen catabolism, positive regulation of intracellular signaling, and regulation of cellular proliferation [39].

In our study, only 2 differentially expressed proteins were detected between OLK and control groups (Figure 2B and Figure S2). These 2 proteins were chemokines, which are involved in many general processes like chemokine-signaling, antimicrobial humoral immune response, and lymphocyte chemotaxis [38].

The differentially expressed proteins between the OLP and the control groups (Figure 2C and Figure S2) were involved in signaling pathway and immune response, being possibly related to the chronic inflammatory background observed in case of OLP [13]. Ten out of 14 proteins were responsible for cell migration, which correlate with the histopathological findings of T-cell and lymphocyte infiltration in the epithelium and lamina propria [38]. Lastly, proteins responsible for positive response to stimulus, stress response, and signaling may reflect the association between viral infection and OLP induction, as well as the association between autoimmune diseases and OLP development [44].

Within the set of proteins that were differentially expressed between OSCC and OLP (Figure 2D and Figure S2), it was eminent that those having known interactions were involved in common biological processes of leukocyte chemotaxis and inflammatory response [45]. This correlates well with the inflammatory background seen in OLP and the infiltration of various leukocyte types in the epithelium and lamina propria, as observed by histopathology [13]. The inflammatory response function might also explain the correlation between systemic autoimmune disease like SLE, RA, and MS with OLP [13]. The majority of the proteins involved connected in the network belong to the cytokine–cytokine receptor interaction KEGG pathway.

The differentially expressed proteins between OSCC and OLK shows a clear and common situation of malignant transformation. Functions related to immune response, cell migration, negative regulation of cell death, collagen catabolism, and the regulation of endopeptidase activity are among the enriched functions (Figure 2F and Figure S2). One key feature was the increase in MMPs expression, which is responsible for the catabolism of collagen in the basement membrane and the ECM disassembly in the connective tissue layer [37]. Another key player is ANXA1, which does not participate in too many interactions, but is involved in the highest number of biological processes [41]. ANXA1 and GPNMB are prominent players in malignant transformation, as both are involved in procarcinogenic biological events, such as negative regulation of cell death and regulation of G1/S transition of mitotic cell cycle. In addition, GPNMB is a marker overexpressed in many other types of cancer (brain, breast, etc.) [36,41]. IL6, a marker shown previously as characteristic to OSCC, is also involved in many inflammatory-related and carcinogenesis-related biological functions [23,28,46].

The proteins that were differentially expressed between the OLP and OLK conditions were few, with no interactions among each other (Figure 2E and Figure S2). In terms of biological processes or molecular function, they were shown by the database to be involved in signaling. No specific common biological functions could be drawn from these unrelated proteins. This further confirmed the understanding that OLK and OLP are two unrelated conditions which separately progress to OSCC [24,25].

**Figure 3:**
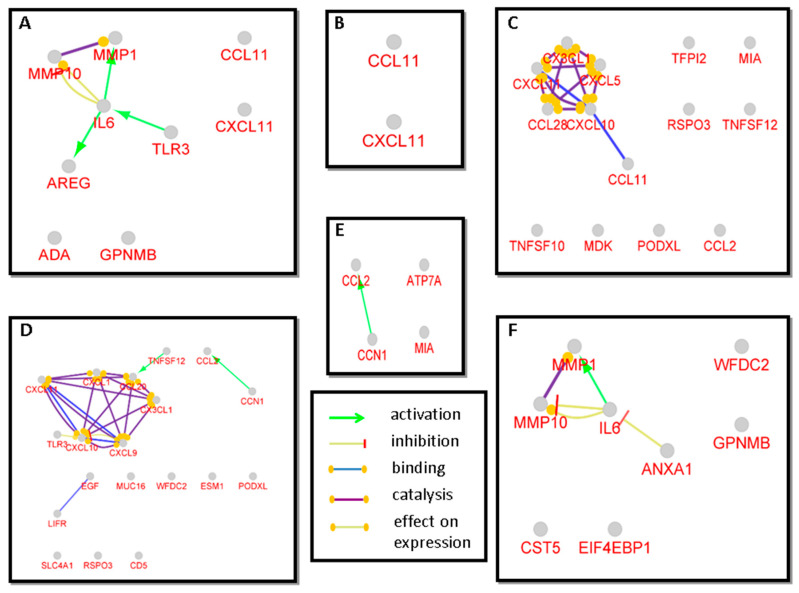
Gene function analysis of the genes corresponding to proteins, with significantly different expression between the patient groups. Each figure represents a gene interaction network (GIN) generated by Cluepedia v1.5.7. Each circle represents a gene and the lines represent the different types of interaction between the genes. The panels show the GIN of the genes of the significantly different proteins between (**A**) OSCC and control, (**B**) OLK and control, (**C**) OLP and control group, (**D**) OSCC and OLP, (**E**) OLK and OLP and (**F**) OLK and OSCC.

In order to gain more information on the proteins showing statistically significant differences between the groups, a Cluepedia search was also performed and the type of interactions were examined (Figure 3).

In addition to the information obtained with String network analysis, the Cluepedia analysis revealed some further information. It was observed that in OSCC group compared to control TLR3 and IL1α activate IL6, which in turn activates AREG and MMP1, and inhibits MMP10 (Figure 3A). Considering the network between OLK and OSCC, it was revealed that ANXA1 inhibits IL6, which then activates MMP1 and inhibits MMP10 (Figure 3F). Regarding the other networks, the interactions observed with String analyses were confirmed. In addition to the already known information, in the networks of proteins with statistically significantly difference between OSCC vs. OLP the activation of CCL20 by TNFSF12 and of CCL2 by CCN1 could be observed (Figure 3D). This latter activation was demonstrated for the network between OLK and OLP as well (Figure 3E). The results obtained by the examination of gene interaction networks confirmed our previous results regarding the difference between OLK and OLP observed with the analysis of String networks (Figure 2).

The differentially expressed proteins between control and OLK and control and OLP groups, respectively, and between OSCC and OLK or OSCC and OLP groups may serve as potential biomarkers for the detection of the precancerous conditions and for their potential progression to OSCC. In this sense, if validated, the identified proteins can be either diagnostic or prognostic biomarkers in case of OLK, OLP, and OSCC.

### 3.4. Verification of the Results Obtained by PEA-Examination of Salivary IL6 Levels Using ELISA

**Figure 4:**
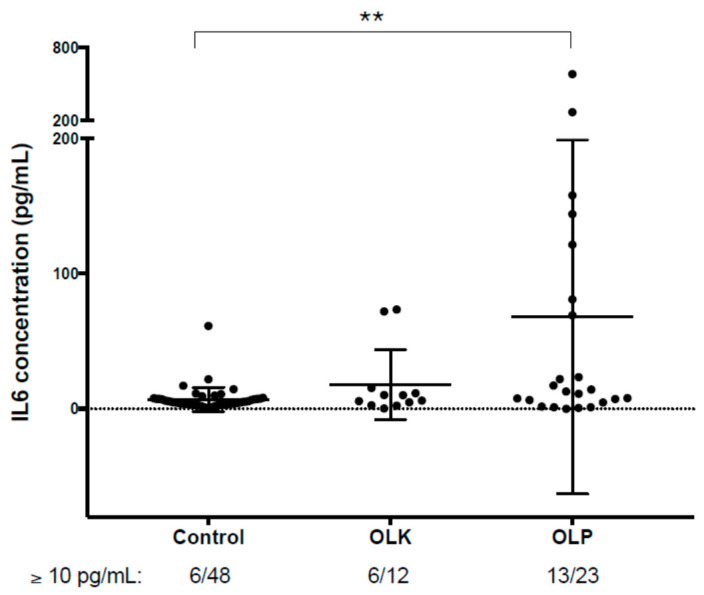
The level of salivary IL6 detected with ELISA. The level of IL6 in saliva samples originating from controls, patients with OLP, patients with OLK was determined using sandwich ELISA. The “*y*” axis shows the concentration of salivary IL6 in case of patient groups presented on the “*x*” axis. The results plotted for the control groups were presented in a previous publication [28]. The stars refer to statistically significant differences: ** *p* < 0.005. The numbers below the *x* axis indicate the number of samples/all samples in each group with IL6 levels above the typical IL6 serum levels (10 pg/mL).

The PEA was carried out on relatively small sample size (3 patients for each group) in order to get a global view on the protein changes characteristic to the precancerous and cancerous conditions, respectively. We were aware that in our experimental setup the results of the PEA analysis can be considered only as a starting point for further experiments. In order to test our results, we have used saliva samples collected from a larger patient cohort (12 patients with OLK and 23 patients with OLP), and the level of IL6 was examined using sensitive sandwich ELISA. IL6 was chosen, because it is involved in inflammatory processes and is a good marker of OSCC, but its effectiveness in the identification of premalignant lesions is controversial [47,48,49,50,51]. We have examined IL6 in our previous study and we have shown that both the mRNA and protein levels of IL6 are elevated in the saliva of patients with OSCC compared to controls [23,28]. In the recent study using PEA, we could observe the higher levels of IL6 in OLK patients as well compared to controls (Figure 1). At the same time, although inflammation is a dominant feature of OLP, IL6 protein expression was not significantly different between the control and OLP groups. These results prompted us to reexamine the level of IL6 in a larger number patients with OLK or OLP using the more sensitive sandwich ELISA (Figure 4) and to compare the expression levels to the previously characterized control group and OSCC group [28] (Figure S3).

There are different values presented in the scientific literature regarding the level of serum IL6 in the normal population, but the typical IL6 concentration measured in serum is below 10 pg/mL in most of the studies [52,53,54]. Compared to the values measured in blood (plasma or serum) higher salivary IL6 levels have been detected [55,56]. In a study applying similar experimental setup as we did, the mean salivary IL6 concentration was found to be 16.8 pg/mL [57], but a larger number of publications show that salivary IL6 concentration is usually less than 10 pg/mL [56,58,59]. Considering the variations in sample collection, methods used for the determination of IL6 levels and the number of publications showing the less than 10 pg/mL value characteristic for saliva of nondiseased controls, we have chosen 10 pg/mL as a threshold for “normal” IL6 protein levels in our study. In the OSCC group 58%, in the OLP group 55%, in the OLK group 50%, while in the control group 12.5% of the patients have shown salivary IL6 values higher than 10 pg/mL (Figure S3). It is clear that in the control group, the percentage of patients whose salivary IL6 concentration is above 10 pg/mL is a minority, while the situation in pathological groups shows a stark contrast. The high number of patients with high IL6 levels in the OLP group fits in with the chronic inflammatory background of the disease [16] and the ratio of the OSCC group is fittingly the highest of all, representing the highly inflammatory condition serving as a basis for cancer [4]. The more sensitive sandwich ELISA, but not the PEA method captured the higher IL6 expression in OLP, as expected.

We do not have any further information regarding the controls with salivary IL6 expression above the 10 pg/mL threshold. At the time of sample collection and medical examination, the patients did not show any signs of localized inflammation in their oral cavity, however, the presence of asymptomatic preclinical conditions involving some form of low-grade but widespread inflammation cannot be excluded. This can be a reasonable explanation in the light of the fact that the oral hygiene in the age-matched controls was relatively poor.

### 3.5. Examination of Salivary IL6 mRNA Levels in Patients with Precancerous Oral Lesions

**Figure 5:**
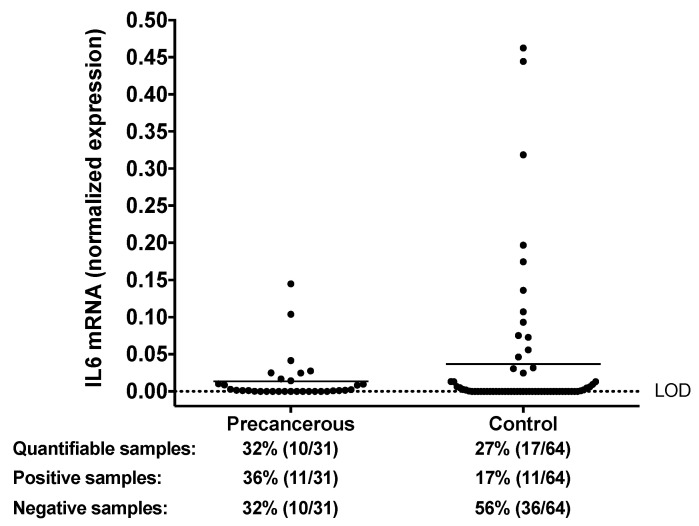
Salivary *IL6* mRNA levels detected by RT-qPCR. Normalized salivary *IL6* mRNA expression in patients with precancerous conditions (OLK or OLP) and in controls. The horizontal lines indicate the mean. Definition of *IL6*-quantifiable, positive and negative samples is provided in the Materials and Methods. The dotted line indicates the limit of detection (LOD, negative samples).

Along with the examination of IL6 protein, the *IL6* mRNA was also studied. In our previous analysis, the salivary *IL6* mRNA levels were quantified in controls and patients with OSCC, and significantly higher *IL6* mRNA levels were observed in the saliva of cancer patients [28]. Compared to patients with OSCC, few patients with precancerous conditions (OLK or OLP) had reliably quantifiable levels of *IL6* mRNA in their saliva. On the other hand, there appears to be an upward trend when compared to control patients: there are more precancerous patients with reliably quantifiable levels of *IL6* mRNA (32% vs. 27%), or with borderline *IL6* positivity (36% vs. 17%) (Figure 5). This suggests that elevated salivary *IL6* mRNA levels may be specifically associated with neoplastic transformation in the oral cavity.

Although more patients with OLP than OLK had quantifiable levels of *IL6* mRNA in their saliva (9 OLP vs. 1 OLK), the small sample size for each precancerous condition does not allow reliable differentiation between them regarding their *IL6* mRNA status.

As indicated in supplementary Table S1, all the samples used for ELISA analysis were used for mRNA analysis as well. We did not find a correlation between salivary IL6 protein and mRNA levels derived from the same patient (data not shown). We speculate that the IL6 protein we detected was released from the cells of the oral cavity as an active cytokine, but we do not have exact information on the origin of mRNA. The saliva processing protocol utilized in this study produced a cell-free fraction, and the *IL6* mRNA we detected was most likely isolated from exosome-like vesicles present in the saliva, and not from intact cells. The biological role of various RNAs enclosed in extracellular vesicles in different body fluids is still not fully understood, but they appear to be actively selected and transported by the cells into the vesicles [60,61]. Therefore, the RNA composition of these extracellular vesicles is not representative of the RNA composition of the cytoplasm, removing the correlation between *IL6* mRNA and protein levels in the saliva.

In spite of the observation that the mRNA levels are not necessarily in accordance with protein levels in the biological systems [62,63], our data indicate similar trends in case of *IL6* mRNA and protein levels among the studied groups. The cause of IL6 protein and mRNA positivity in the control group (12.5 and 17%, respectively) has not yet been identified; repeated sampling and measurements, and follow-up clinical examinations over a longer time period may reveal the biological processes behind it. Similarly, a longer follow-up of precancerous patients may reveal the changes of salivary IL6 protein and mRNA levels over time, and also whether increasing salivary IL6 levels are associated with the progression to the cancerous state in the same patient.

## 4. Conclusions

Proximity extension assay and network analysis of the results was carried out in case of saliva samples originating from controls, patients with OSCC, OLP, or OLK. We could observe proteins whose level changed in a statistically significant manner between the examined groups. As expected, in the OSCC group proteins related to carcinogenesis were shown to be expressed differentially, while the network analysis of the significantly different proteins indicated the inflammation as the biological processes characteristic for OLP and the malignant transformation as characteristic for OLK. These data further demonstrate the different nature of OLK and OLP, even if they can both progress to OSCC. IL6 expression was examined both at the protein and the mRNA level, and the differences among the disease groups could be demonstrated. Our results can serve as starting point for further studies involving not only the OSCC but the precancerous lesions as well in order to better understand the premalignant—malignant transformation and to validate biomarkers for OSCC, OLK, and OLP.

## Figures and Tables

**Table 1:** Patient data. The sex, age, and gingival index in case of patient groups along with the alcohol and smoking habits. OLP=oral lichen planus, OLK=oral leukoplakia.

		OLP	OLK
		(N = 23)	(N = 12)
Sex	Male (N, %)	9 (40%)	11 (33%)
	Female (N, %)	14 (60%)	22 (66%)
Age	(years; mean ± SD)	62 ± 3.5	62 ± 5
Gingival index (Loe–Silness index)	(mean ± SD)	0.56 ± 0.35	0.13 ± 0.06
Regular ethanol consumption	Number of regular alcohol consumers, %	4 (17%)	5 (41%)
Smoking	Number of smokers, %	5 (22%)	2 (16%)

N = number of patients.

## Supplementary Materials

Supplementary materials can be found at https://www.mdpi.com/2227-9059/8/12/610/s1. Table S1. Patient data. The table lists each patient included in the present study, with the disease state and with the type of analysis performed on the saliva samples specified. Table S2. Proteins in the Olink panels. The protein name, protein code, Uniprot ID, and Olink ID along with the name of the panel is given for each analyzed protein. Table S3. Raw data obtained by proximity extension assay. The normalized protein expression (NPX) values corresponding for the examined proteins for each individual sample are presented. Values below limit of detection (LOD) are highlighted with red background and the LOD value is given. In case of the examined proteins, the protein code, the Uniprot ID, and the Olink ID is provided along with data corresponding to the panel used. In case of each protein the LOD, missing data frequency and the detectability is indicated as well. Figure S1. The amount of proteins having statistically significant changes between at least two groups. The NPX values for each protein presented on Figure 1 (*y* axis) in case of saliva samples originating from controls, patients with OLP, patients with OLK, and patients with OSCC (*x* axis) are shown. The table showing the p values is also included. The highlighted values show *p* < 0.05. Figure S2. The enriched GO functions according to String in case of networks presented in Figure 2. Figure S3. The level of salivary IL6 detected with ELISA. The level of IL6 in saliva samples originating from controls, patients with OLP, patients with OLK, and patients with OSCC was determined using sandwich ELISA. The “*y*” axis shows the concentration of salivary IL6 in case of patient groups presented on the “*x*” axis. The results plotted in case of control and OSCC groups were presented in a previous publication [28].The stars refer to statistically significant differences: ** *p* < 0.005, *** *p* = 0.0001. The numbers below the *x* axis indicate the number of samples/all samples in each group with IL6 levels above the typical IL6 serum levels (10 pg/mL).
