# Supplementary material for: Examination of Oral Squamous Cell Carcinoma and Precancerous Lesions Using Proximity Extension Assay and Salivary RNA Quantification
Source: Biomedicines. 2020 Dec 14;8(12):610. doi: 10.3390/biomedicines8120610 (PMC7764999; doi:10.3390/biomedicines8120610)
Supplement: Supplementary file 1 [file biomedicines-08-00610-s001.zip › FigureS1.pdf]

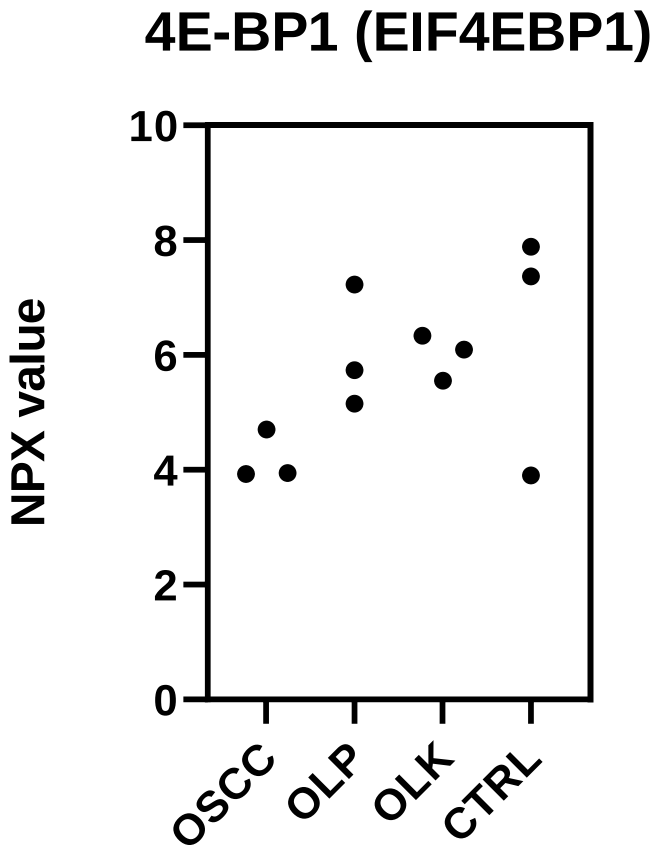

4E-BP1 (EIF4EBP1)
NPX value
10
8
6
4
2
0
OSCC
OLP
OLK
CTRL

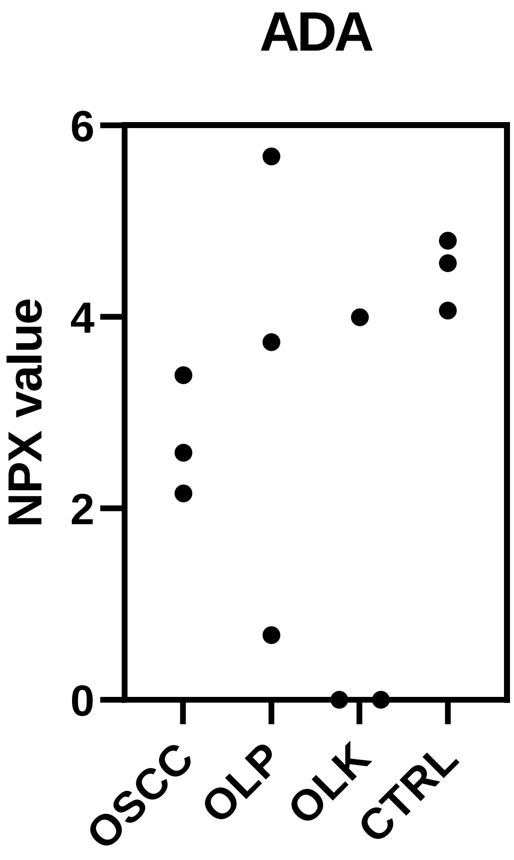
ADA
NPX value
6
4
2
0
OSCC
OLP
OLK
CTRL

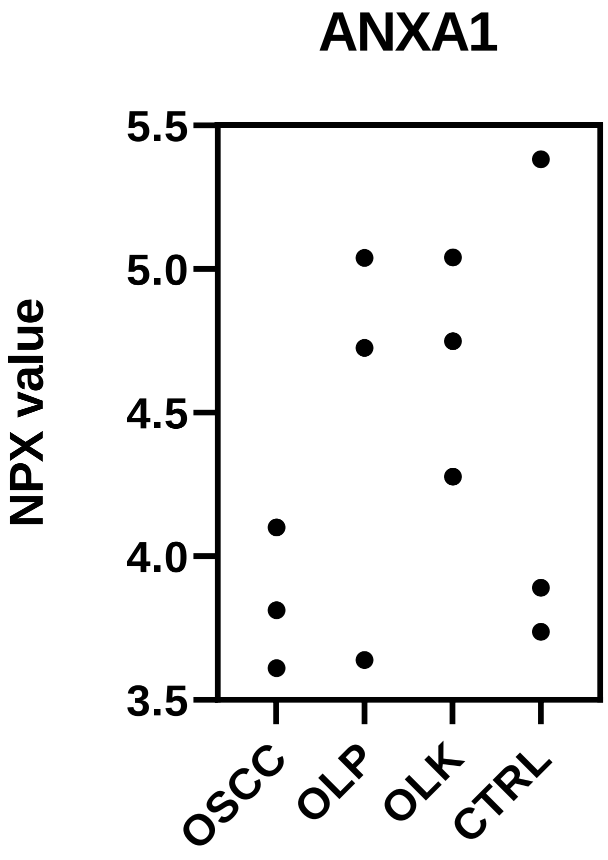
ANXA1
NPX value
5.5
5.0
4.5
4.0
3.5
OSCC
OLP
OLK
CTRL

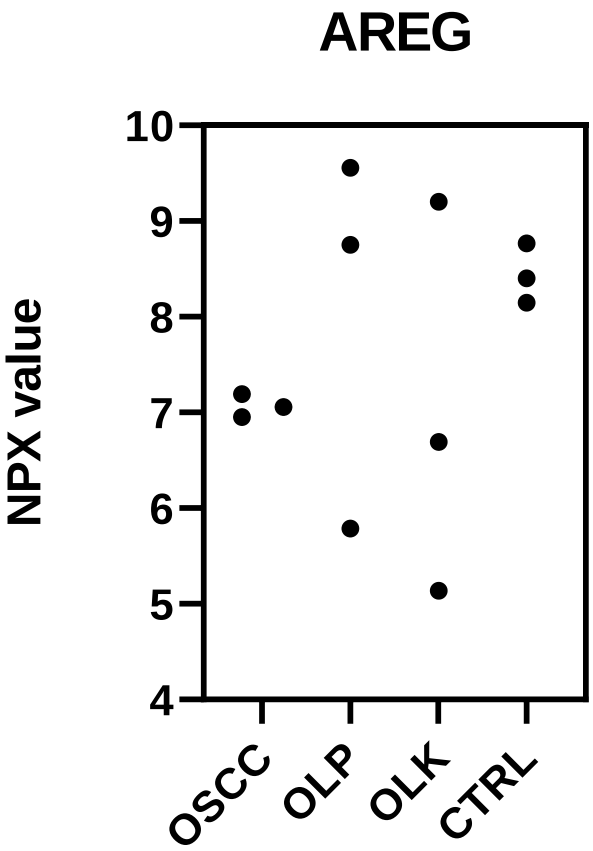
AREG
NPX value
10
9
8
7
6
5
4
OSCC
OLP
OLK
CTRL

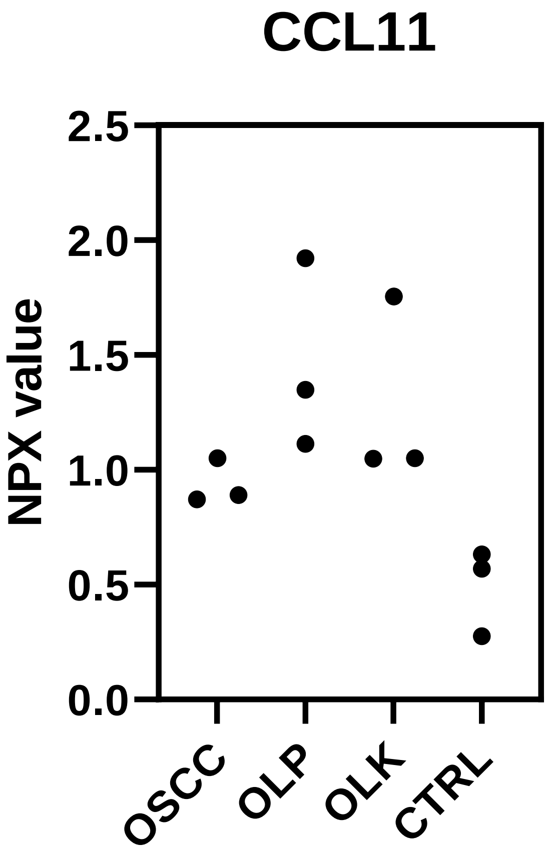
CCL11
NPX value
2.5
2.0
1.5
1.0
0.5
0.0
OSCC
OLP
OLK
CTRL

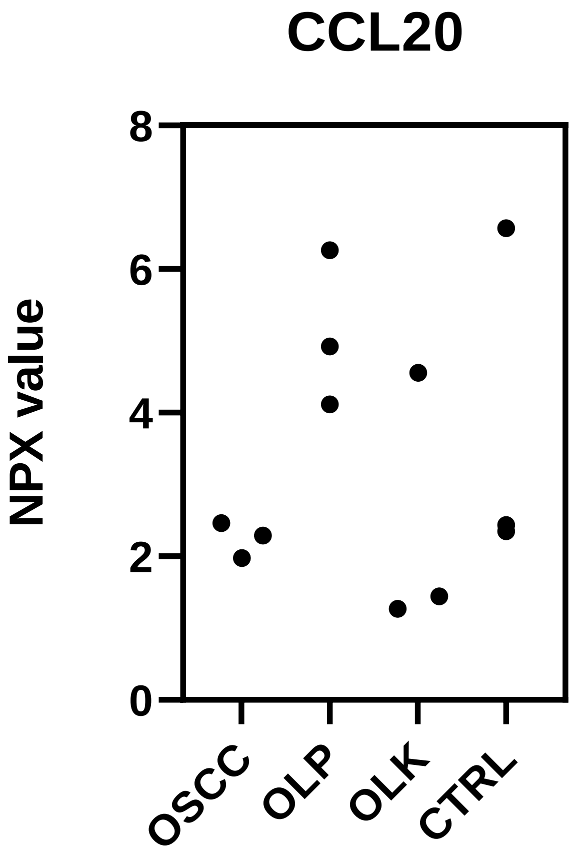
CCL20
NPX value
8
6
4
2
0
OSCC
OLP
OLK
CTRL

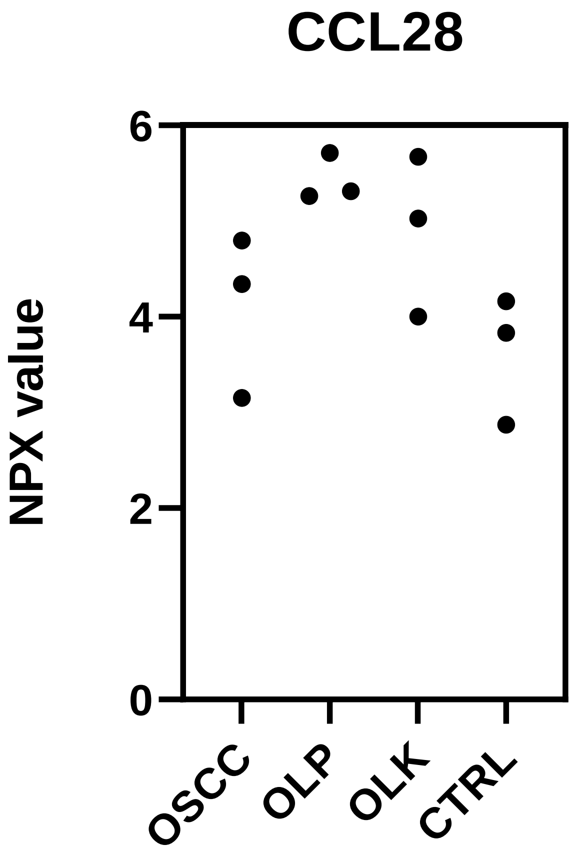
CCL28
NPX value
6
4
2
0
OSCC
OLP
OLK
CTRL

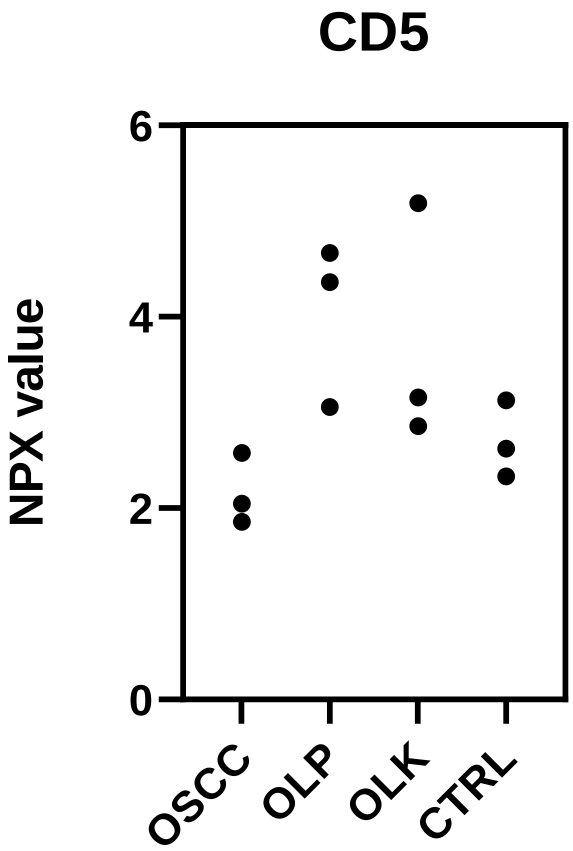
CD5
NPX value
6
4
2
0
OSCC
OLP
OLK
CTRL

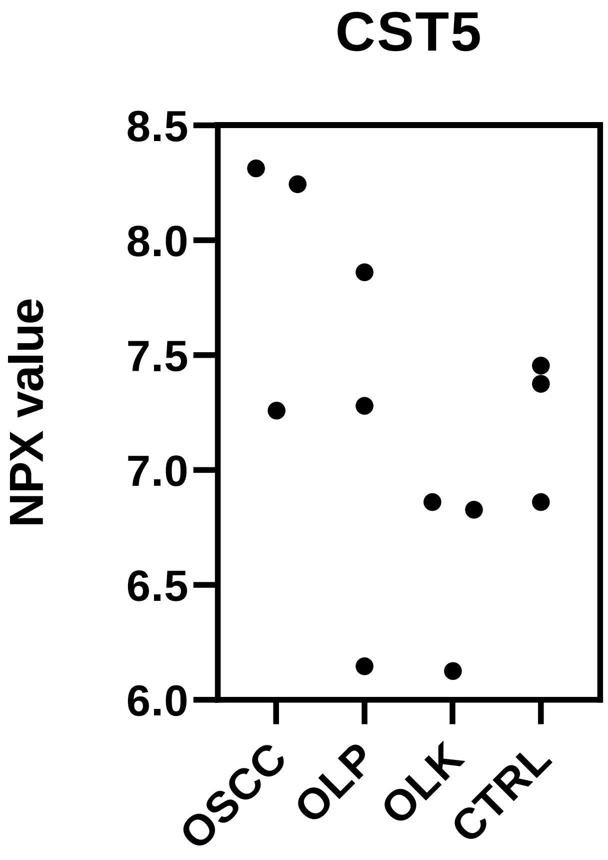

CST5
NPX value
8.5
8.0
7.5
7.0
6.5
6.0
OSCC
OLP
OLK
CTRL

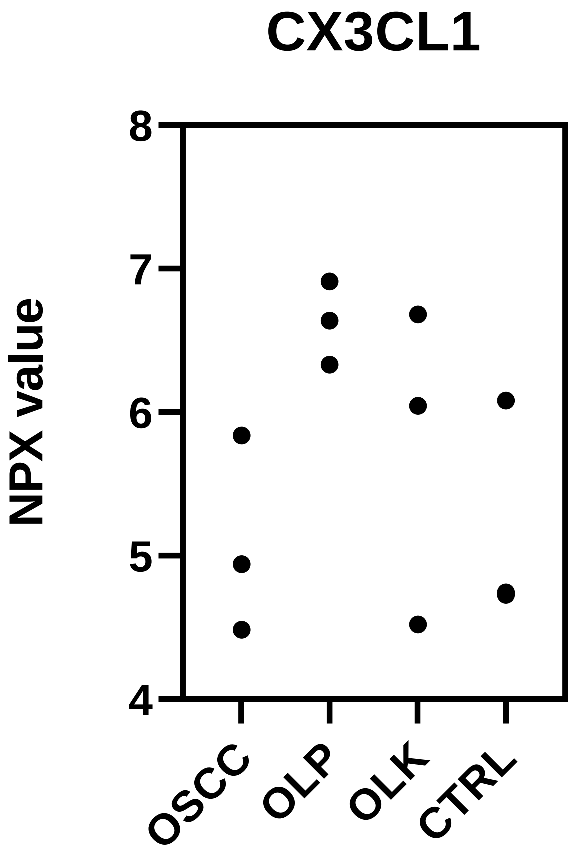
CX3CL1
NPX value
8
7
6
5
4
OSCC
OLP
OLK
CTRL

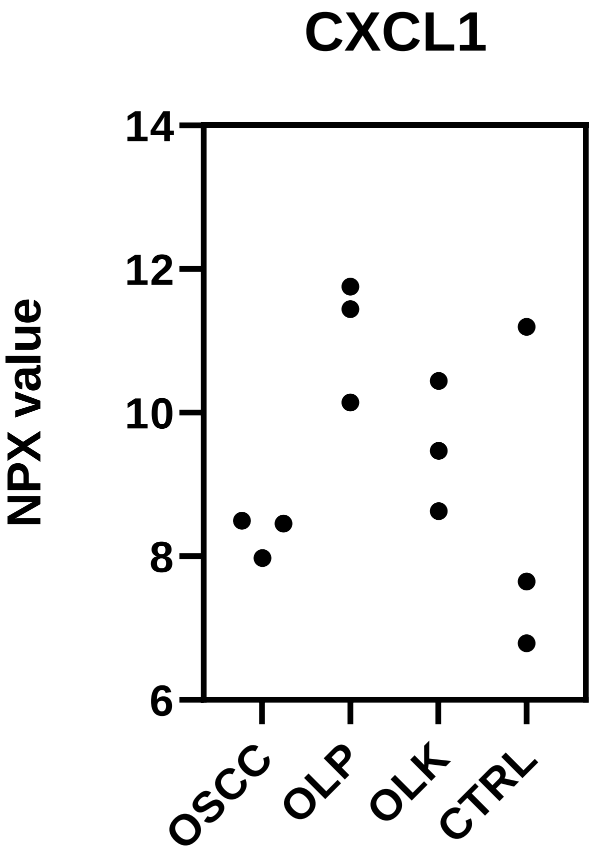
CXCL1
NPX value
14
12
10
8
6
OSCC
OLP
OLK
CTRL

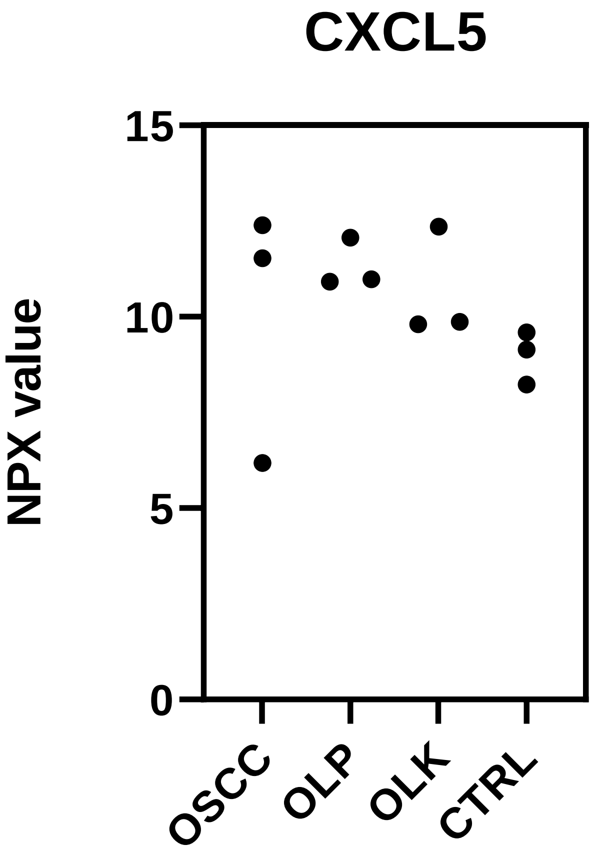

CXCL5
NPX value
15
10
5
0
OSCC
OLP
OLK
CTRL

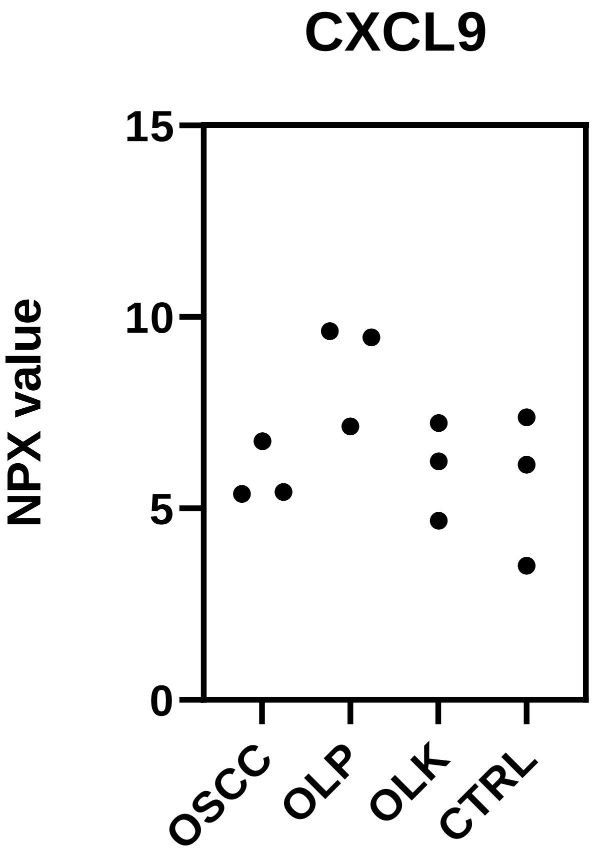
CXCL9
NPX value
15
10
5
0
OSCC
OLP
OLK
CTRL

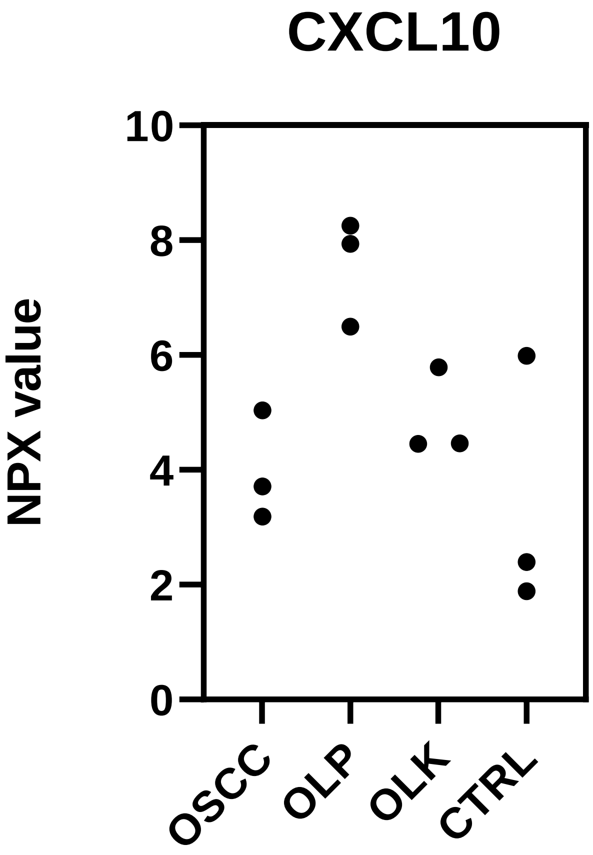
CXCL10
NPX value
0
2
4
6
8
10
OSCC
OLP
OLK
CTRL

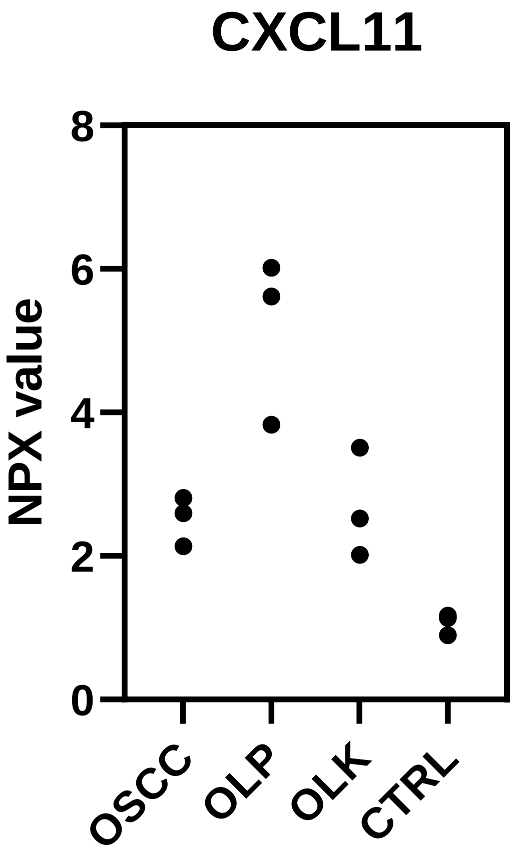
CXCL11
NPX value
8
6
4
2
0
OSCC
OLP
OLK
CTRL

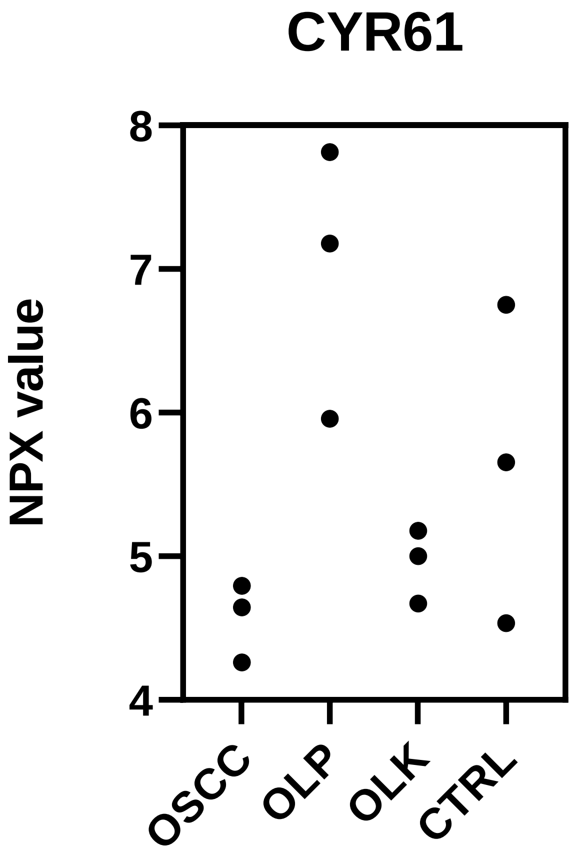
CYR61
NPX value
8
7
6
5
4
OSCC
OLP
OLK
CTRL

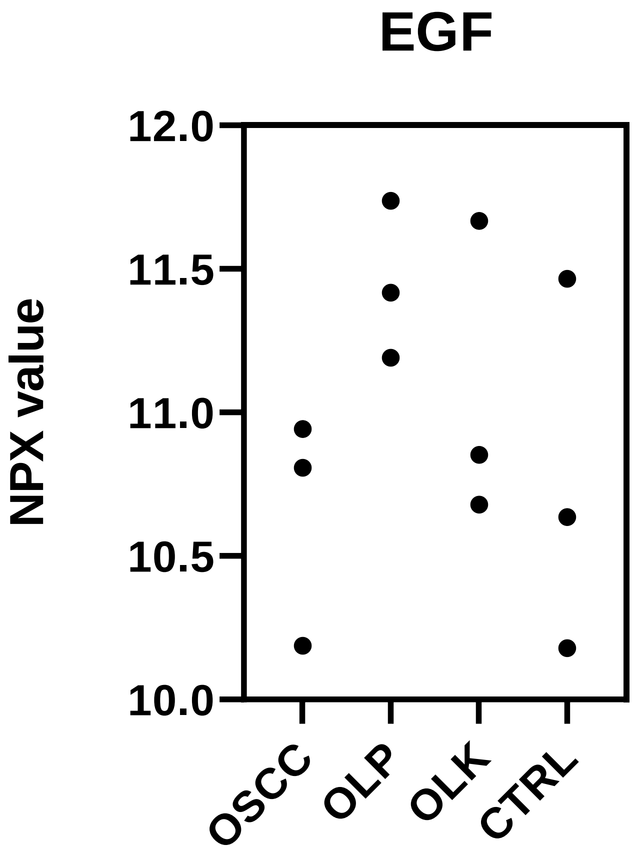
EGF
NPX value
12.0
11.5
11.0
10.5
10.0
OSCC
OLP
OLK
CTRL

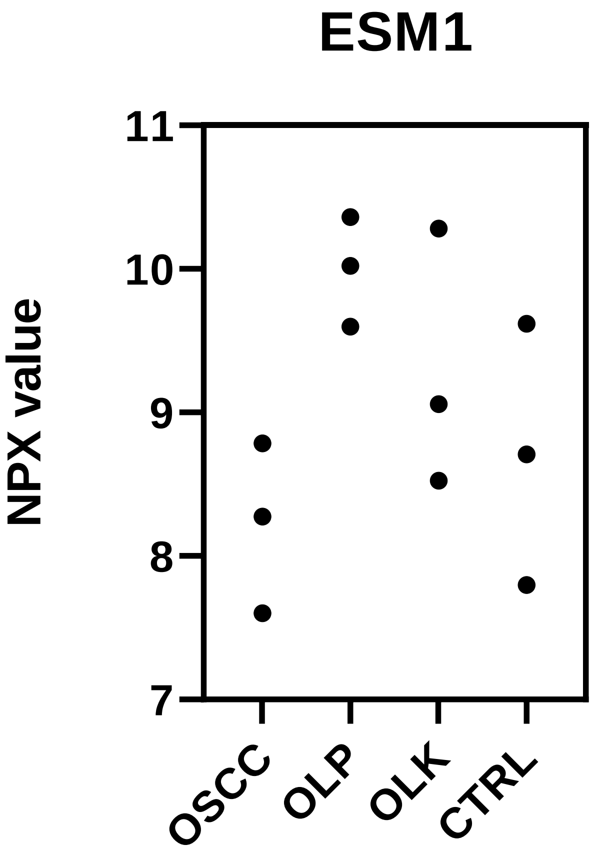
ESM1
NPX value
11
10
9
8
7
OSCC
OLP
OLK
CTRL

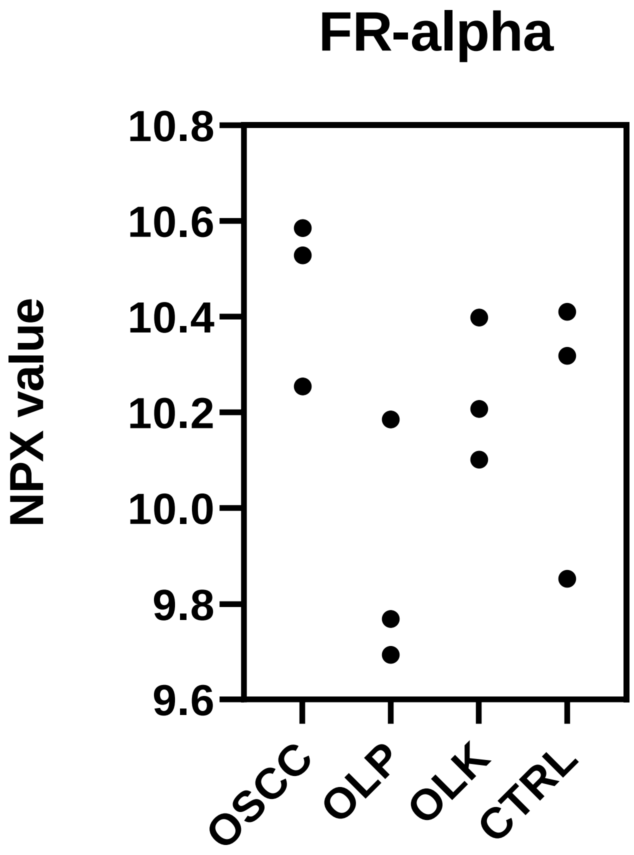
FR-alpha
NPX value
10.8
10.6
10.4
10.2
10.0
9.8
9.6
OSCC
OLP
OLK
CTRL

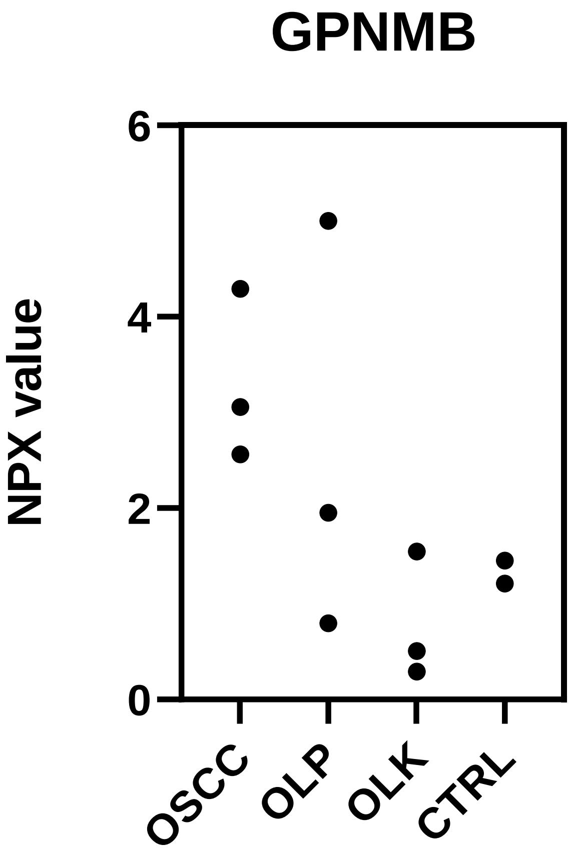
GPNMB
NPX value
6
4
2
0
OSCC
OLP
OLK
CTRL

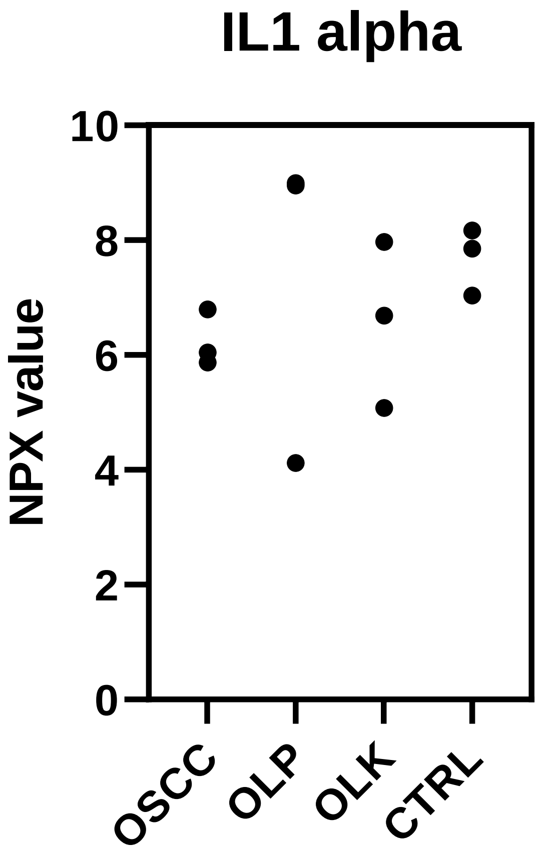
IL1 alpha
NPX value
10
8
6
4
2
0
OSCC
OLP
OLK
CTRL

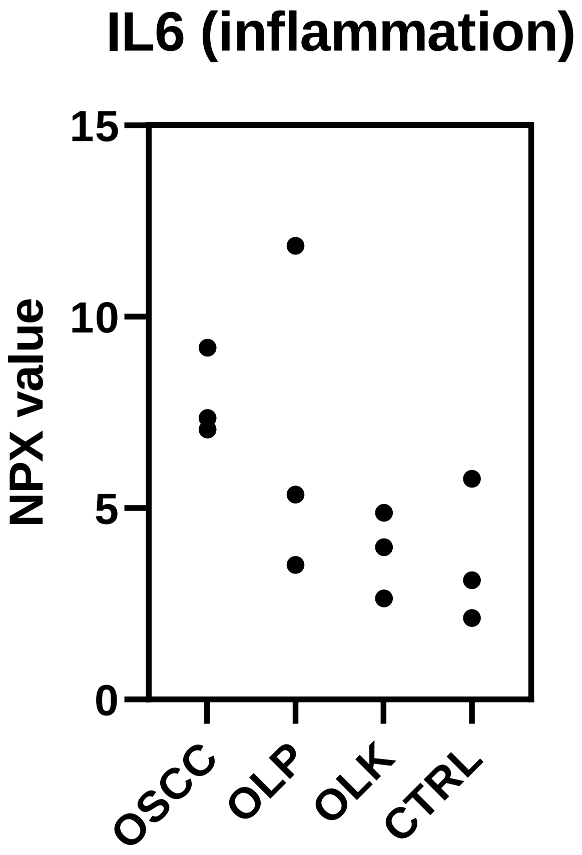

IL6 (inflammation)
NPX value
15
10
5
0
OSCC
OLP
OLK
CTRL

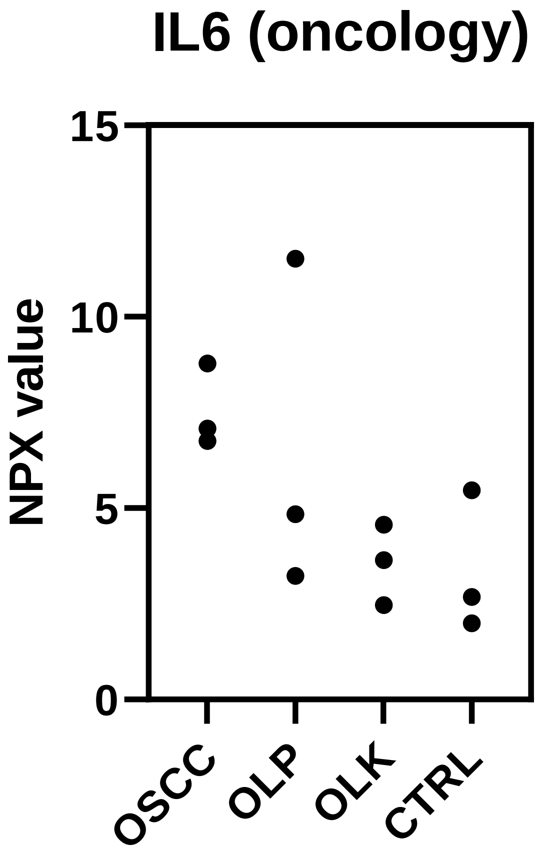
IL6 (oncology)
NPX value
15
10
5
0
OSCC
OLP
OLK
CTRL

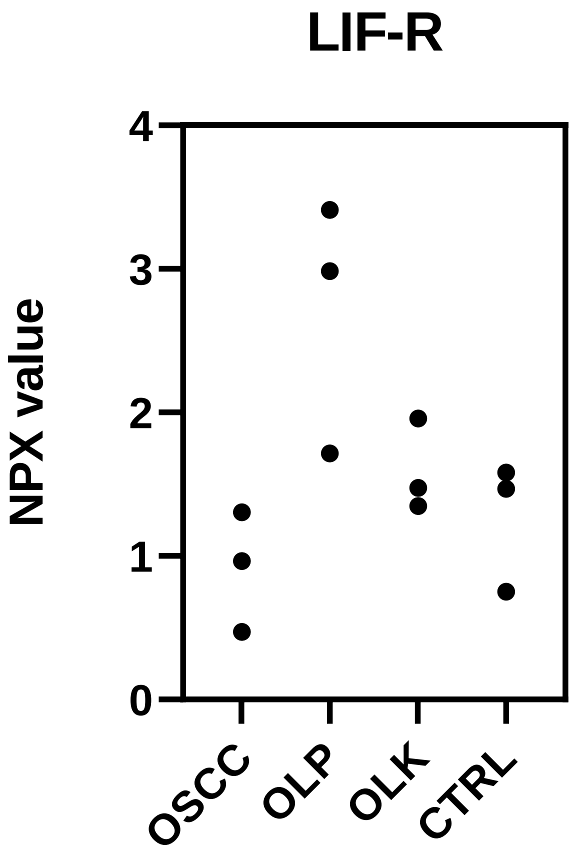

LIF-R
NPX value
0
1
2
3
4
OSCC
OLP
OLK
CTRL

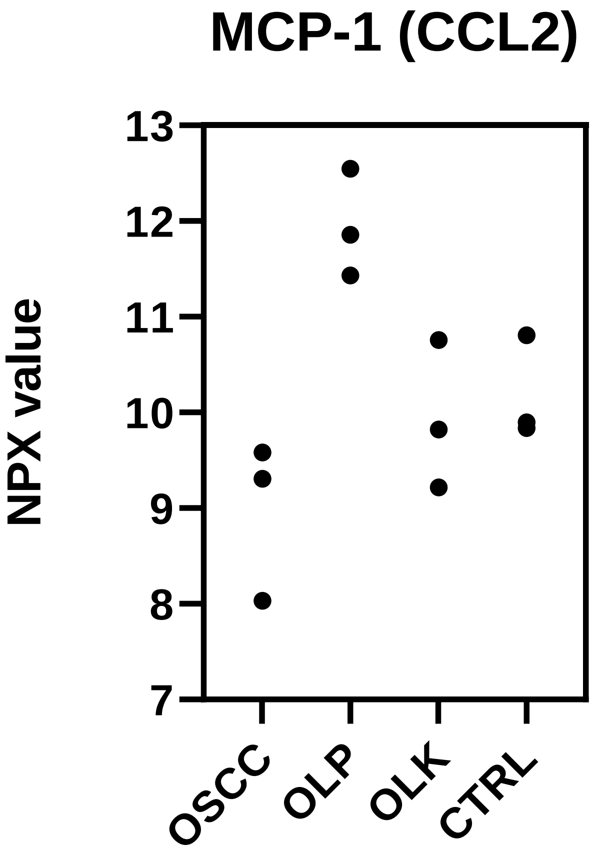

MCP-1 (CCL2)
NPX value
13
12
11
10
9
8
7
OSCC
OLP
OLK
CTRL

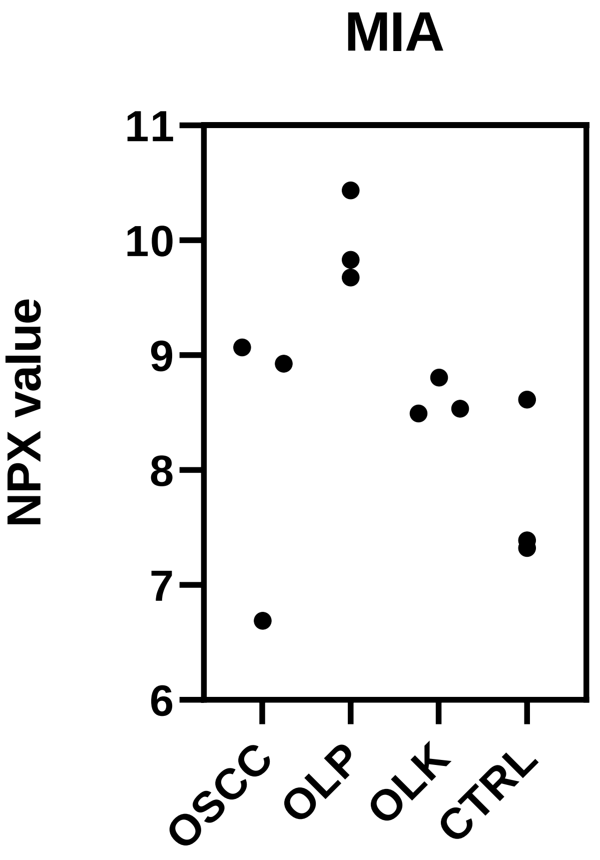
MIA
NPX value
11
10
9
8
7
6
OSCC
OLP
OLK
CTRL

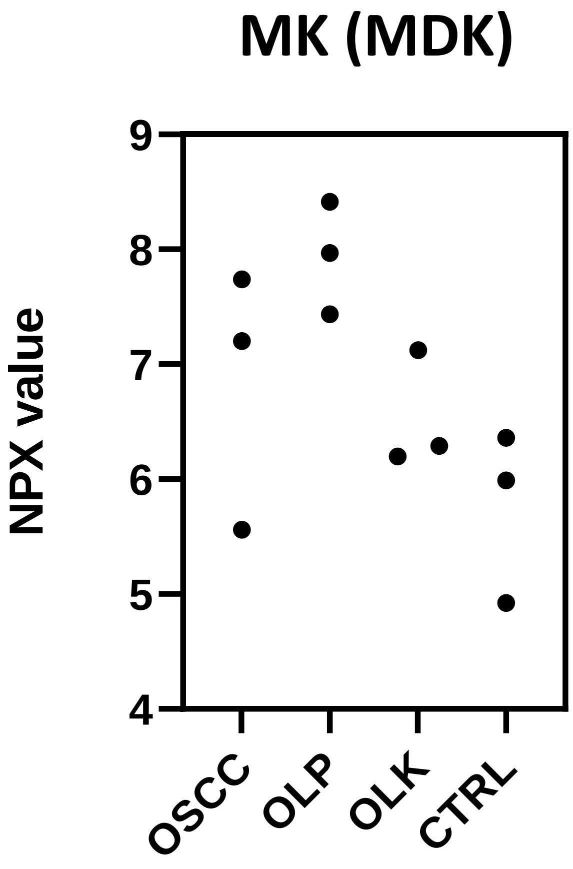
MK (MDK)
NPX value
9
8
7
6
5
4
OSCC
OLP
OLK
CTRL

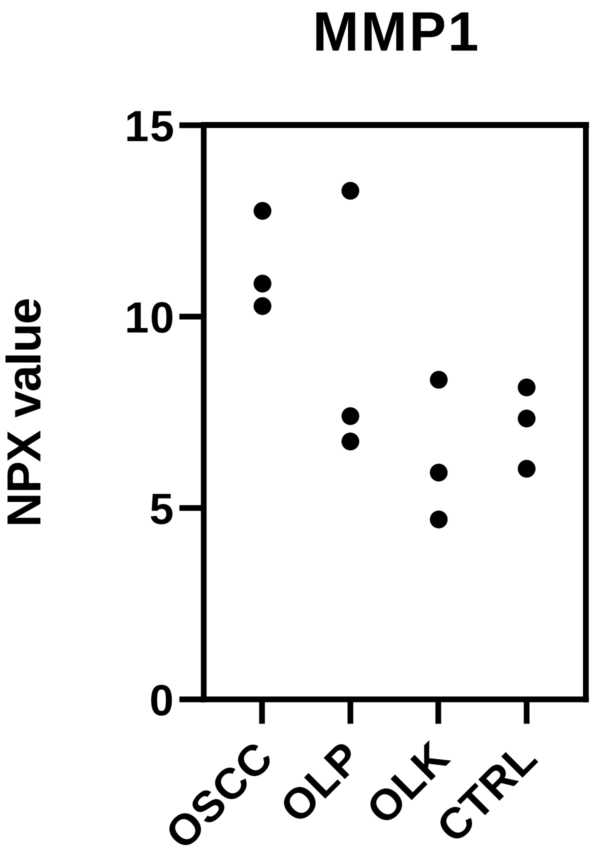
MMP1
NPX value
15
10
5
0
OSCC
OLP
OLK
CTRL

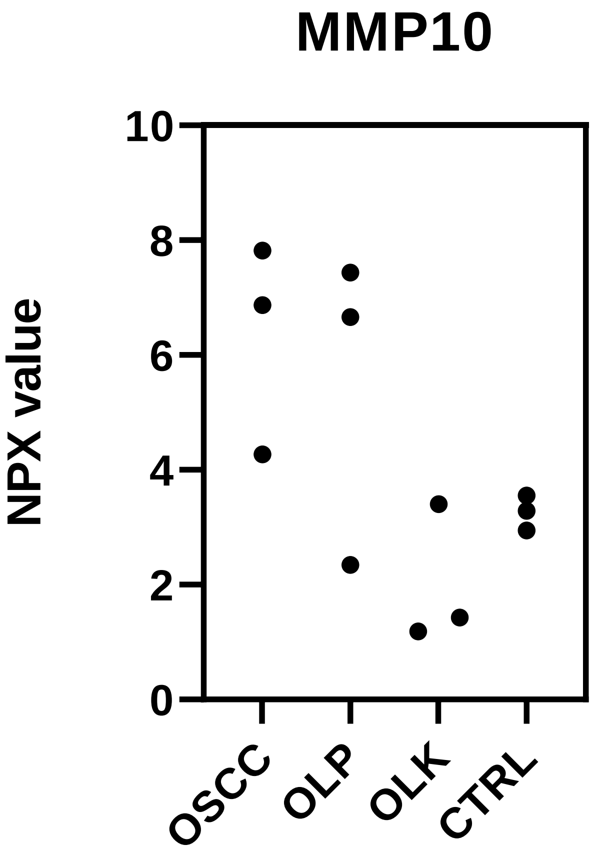
MMP10
NPX value
10
8
6
4
2
0
OSCC
OLP
OLK
CTRL

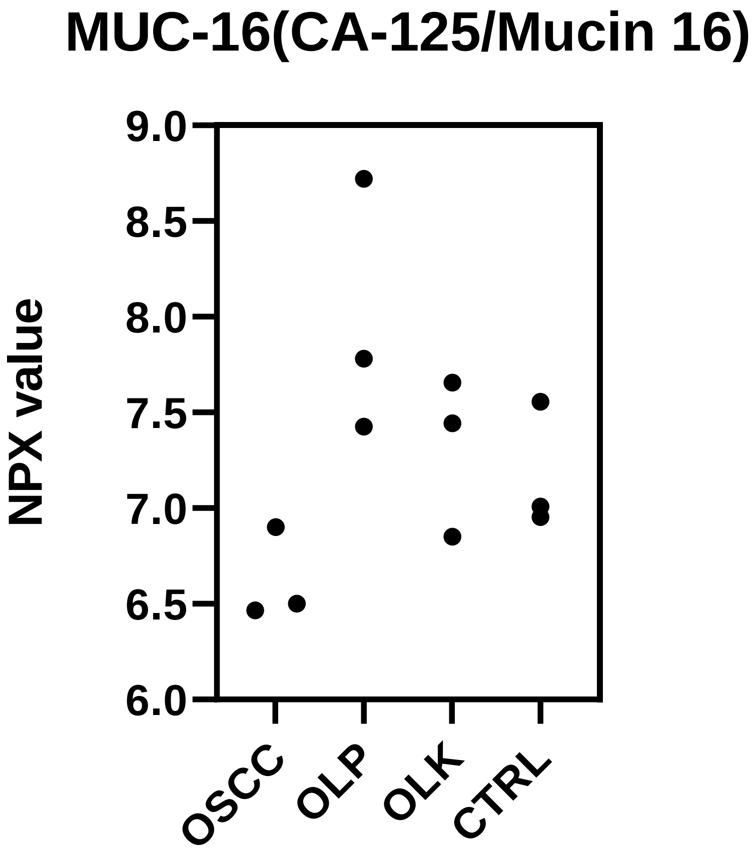

MUC-16(CA-125/Mucin 16)
NPX value
9.0
8.5
8.0
7.5
7.0
6.5
6.0
OSCC
OLP
OLK
CTRL

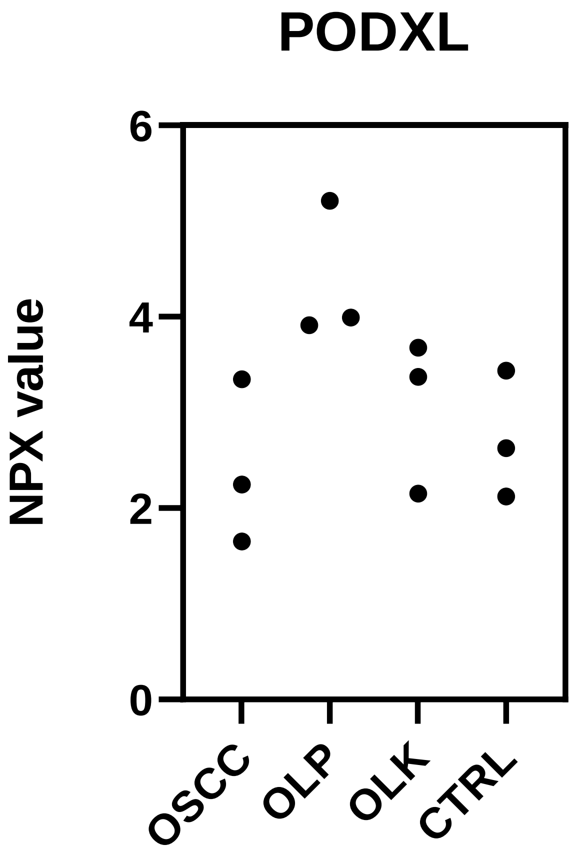
PODXL
NPX value
6
4
2
0
OSCC
OLP
OLK
CTRL

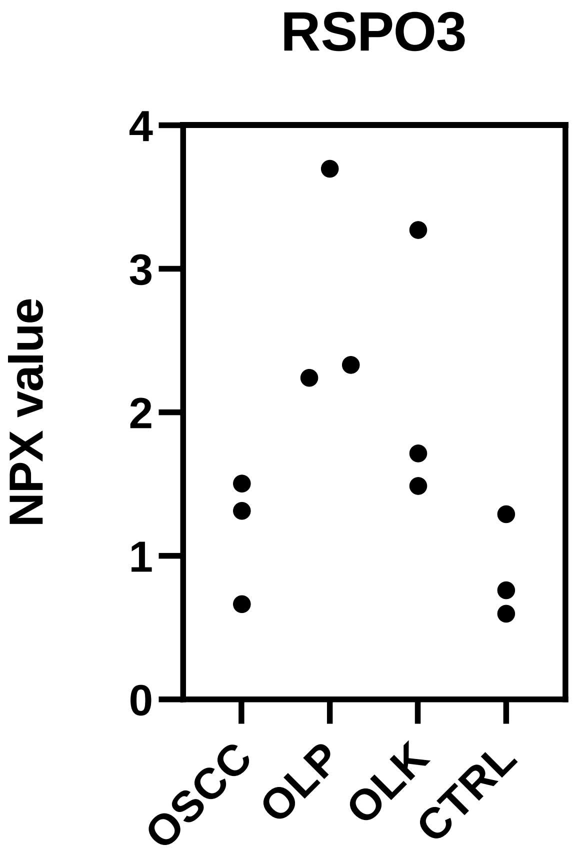
RSPO3
NPX value
0
1
2
3
4
OSCC
OLP
OLK
CTRL

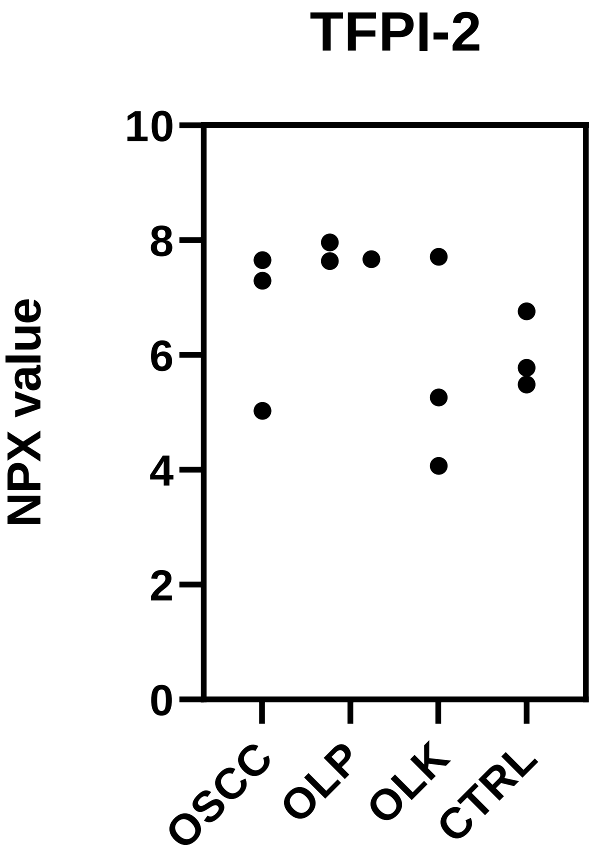

TFPI-2
NPX value
0
2
4
6
8
10
OSCC
OLP
OLK
CTRL

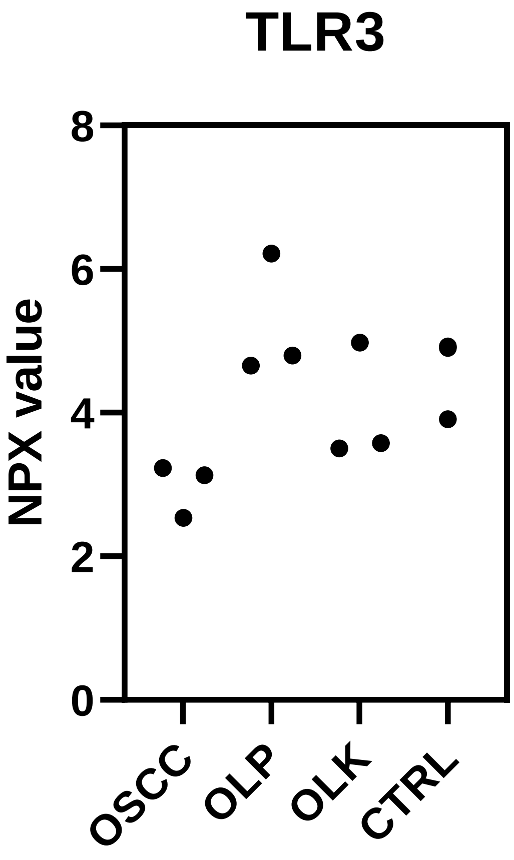
TLR3
NPX value
0
2
4
6
8
OSCC
OLP
OLK
CTRL

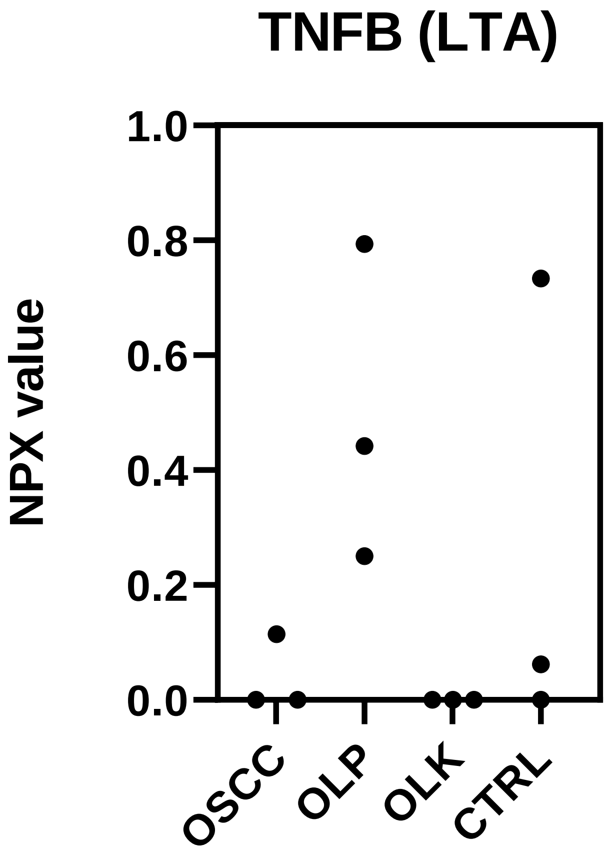
TNFB (LTA)
NPX value
1.0
0.8
0.6
0.4
0.2
0.0
OSCC
OLP
OLK
CTRL

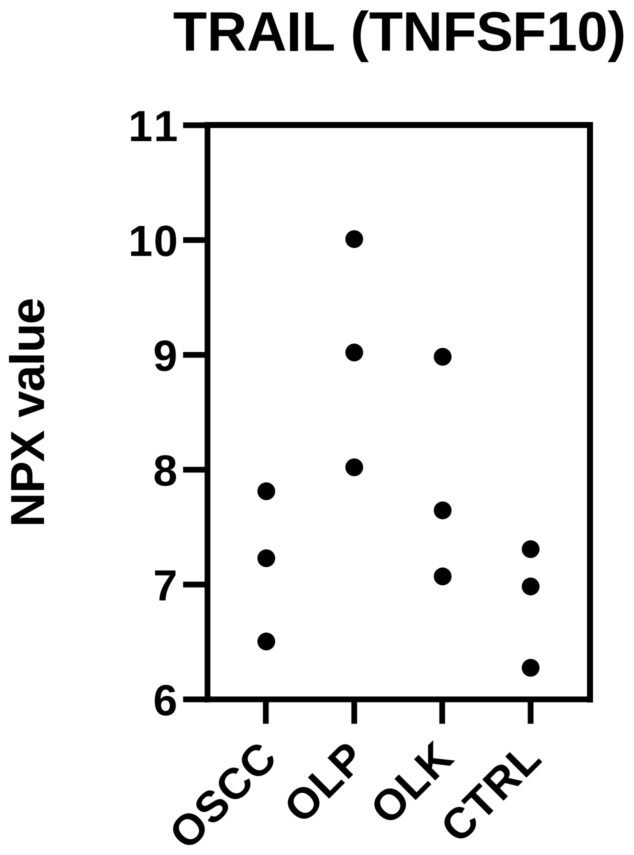
TRAIL (TNFSF10)
NPX value
11
10
9
8
7
6
OSCC
OLP
OLK
CTRL

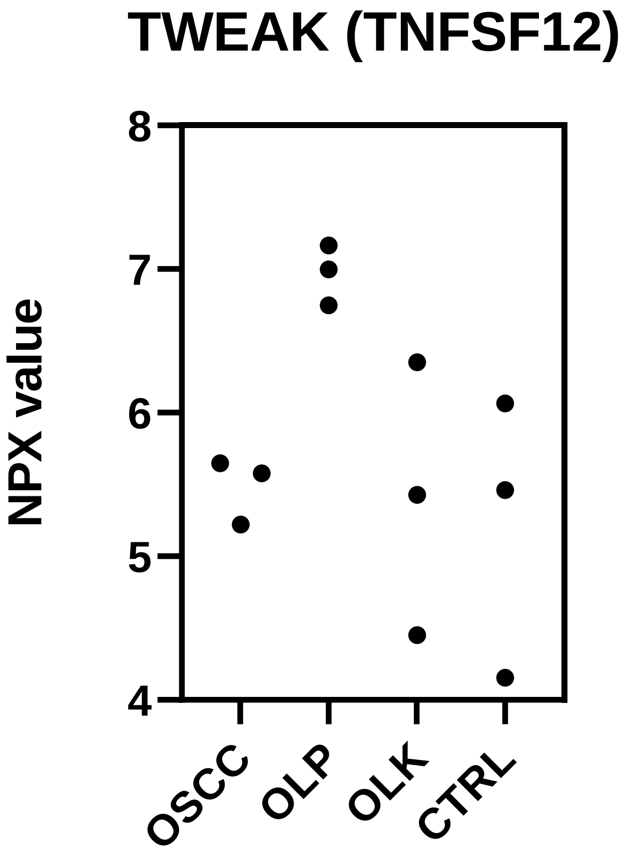
TWEAK (TNFSF12)
NPX value
8
7
6
5
4
OSCC
OLP
OLK
CTRL

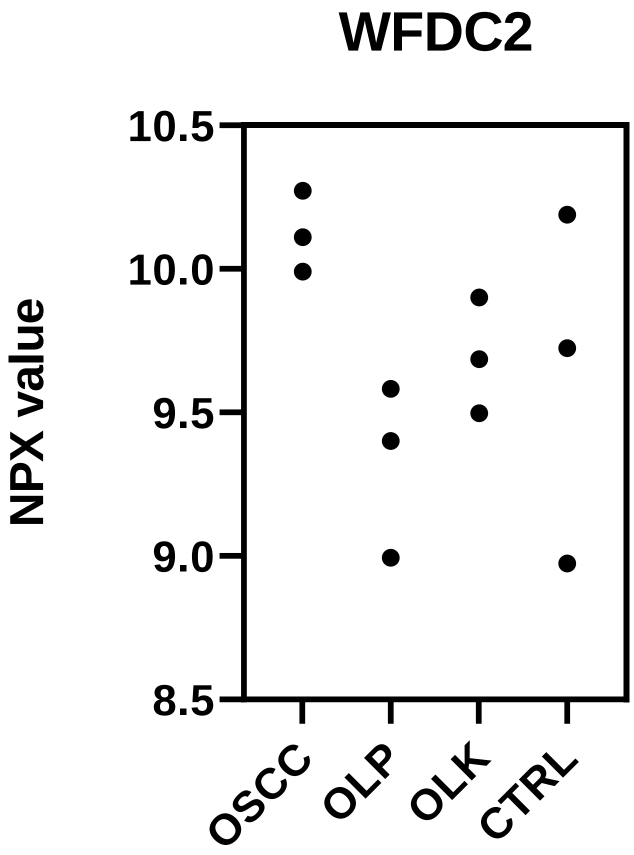
WFDC2
NPX value
10.5
10.0
9.5
9.0
8.5
OSCC
OLP
OLK
CTRL

	OSCC-CTRL	OLP-CTRL	OLK-CTRL	OSCC-OLP	OSCC-OLK	OLP-OLK
4E-BP1	0,218	0,816	0,785	0,051	0,006	0,947
ADA	0,014	0,493	0,139	0,685	0,375	0,362
ANXA1	0,413	0,856	0,569	0,234	0,032	0,667
AREG	0,002	0,758	0,298	0,489	0,964	0,569
CCL11	0,023	0,021	0,038	0,101	0,275	0,628
CCL20	0,382	0,438	0,480	0,011	0,881	0,097
CCL28	0,488	0,012	0,108	0,059	0,309	0,355
CD5	0,167	0,071	0,248	0,026	0,108	0,755
CX3CL1	0,880	0,040	0,510	0,024	0,430	0,256
CXCL1	0,879	0,148	0,540	0,006	0,094	0,090
CXCL10	0,713	0,042	0,341	0,010	0,262	0,019
CXCL11	0,003	0,024	0,023	0,020	0,748	0,037
CXCL5	0,647	0,013	0,145	0,578	0,779	0,518
CXCL9	0,893	0,093	0,800	0,035	0,836	0,069
CYR61	0,177	0,187	0,348	0,013	0,154	0,023
CST5	0,142	0,815	0,109	0,238	0,033	0,429
EGF	0,809	0,167	0,562	0,046	0,334	0,328
ESM-1	0,480	0,087	0,477	0,012	0,162	0,279
FR-alpha	0,261	0,249	0,837	0,036	0,176	0,115
GPNMB	0,018	0,413	0,315	0,623	0,017	0,242
IL-1 alpha	0,031	0,860	0,288	0,562	0,719	0,691
IL6 (inflammation)	0,030	0,304	0,902	0,734	0,012	0,304
IL6 (oncology)	0,028	0,316	0,890	0,719	0,011	0,319
LIF-R	0,377	0,066	0,363	0,034	0,089	0,110
MCP-1	0,102	0,017	0,675	0,007	0,216	0,022
MIA	0,633	0,010	0,179	0,095	0,670	0,005
MK	0,242	0,013	0,209	0,196	0,702	0,026
MMP-1	0,013	0,415	0,532	0,384	0,019	0,295
MMP-10	0,047	0,296	0,157	0,683	0,028	0,115
MUC-16	0,082	0,136	0,665	0,030	0,068	0,221
PODXL	0,644	0,045	0,605	0,040	0,393	0,106
RSPO3	0,445	0,022	0,100	0,041	0,181	0,459
TFPI-2	0,514	0,012	0,788	0,312	0,509	0,126
TLR3	0,015	0,342	0,393	0,014	0,117	0,157
TNFB	0,436	0,463	0,376	0,049	0,423	0,036
TRAIL	0,516	0,030	0,165	0,060	0,342	0,251
TWEAK	0,682	0,039	0,827	0,001	0,905	0,050
WFDC2	0,245	0,485	0,869	0,014	0,039	0,153
