# Supplementary material for: Examination of Oral Squamous Cell Carcinoma and Precancerous Lesions Using Proximity Extension Assay and Salivary RNA Quantification
Source: Biomedicines. 2020 Dec 14;8(12):610. doi: 10.3390/biomedicines8120610 (PMC7764999; doi:10.3390/biomedicines8120610)
Supplement: Supplementary file 1 [file biomedicines-08-00610-s001.zip › FigureS2.pdf]

Enriched GO functions for OLP vs. control groups

explain columns

Biological Process (Gene Ontology)

GO-term	description	count in network	strength	false discovery rate
GO:0048246	macrophage chemotaxis	2 of 14	2.3	0.00058
GO:0010818	T cell chemotaxis	2 of 15	2.27	0.00063
GO:0072678	T cell migration	3 of 26	2.21	2.47e-05
GO:1901623	regulation of lymphocyte chemotaxis	2 of 21	2.12	0.0010
GO:0048247	lymphocyte chemotaxis	4 of 45	2.09	1.68e-06

(more ...)

Molecular Function (Gene Ontology)

GO-term	description	count in network	strength	false discovery rate
GO:0048248	CXCR3 chemokine receptor binding	2 of 5	2.75	8.87e-05
GO:0045236	CXCR chemokine receptor binding	3 of 16	2.42	3.00e-06
GO:0008009	chemokine activity	7 of 48	2.31	3.36e-13
GO:0048020	CCR chemokine receptor binding	3 of 41	2.01	3.68e-05
GO:0005164	tumor necrosis factor receptor binding	2 of 31	1.96	0.0018

(more ...)

Cellular Component (Gene Ontology)

GO-term	description	count in network	strength	false discovery rate
GO:0005902	microvillus	2 of 82	1.53	0.0491
GO:0005615	extracellular space	10 of 1134	1.09	1.66e-08
GO:0005576	extracellular region	13 of 2505	0.86	2.91e-09

Reference publications (PubMed)

publication	(year) title	count in network	strength	false discovery rate
PMID:31740970	(2020) Novel associations between cytokines and pulmonar...	3 of 3	3.15	1.31e-06
PMID:9164978	(1997) Expression of IFN-inducible protein-10 in chronic hep...	2 of 2	3.15	8.65e-05
PMID:8903849	(1995) Central nervous system chemokine mRNA accumula...	2 of 2	3.15	8.65e-05
PMID:8472896	(1993) Astrocyte expression of mRNA encoding cytokines I...	2 of 2	3.15	8.65e-05
PMID:33192326	(2020) Phenotyping CCL2 Containing Central Amygdala Neu...	2 of 2	3.15	8.65e-05

(more ...)

local network cluster (STRING)

cluster	description	count in network	strength	false discovery rate
CL:7370	CXCR3 chemokine receptor binding	2 of 5	2.75	4.52e-05
CL:7490	mixed, incl. CCR2 chemokine receptor binding, and CC che...	2 of 7	2.6	7.10e-05
CL:7361	Chemokine receptors bind chemokines	4 of 29	2.29	5.51e-08
CL:7359	Chemokine receptors bind chemokines	5 of 37	2.28	1.87e-09
CL:7364	CXC Chemokine domain, and dendritic cell chemotaxis	3 of 24	2.24	4.21e-06

(more ...)

KEGG Pathways

pathway	description	count in network	strength	false discovery rate
hsa04657	IL-17 signaling pathway	4 of 92	1.78	4.36e-06
hsa04062	Chemokine signaling pathway	7 of 181	1.73	2.73e-10
hsa04668	TNF signaling pathway	4 of 108	1.71	6.07e-06
hsa04060	Cytokine-cytokine receptor interaction	9 of 263	1.68	7.95e-13
hsa05323	Rheumatoid arthritis	2 of 84	1.52	0.0070

(more ...)

Enriched GO functions for OLK vs. control groups

explain columns

Biological Process (Gene Ontology)

GO-term	description	count in network	strength	false discovery rate
GO:0048247	lymphocyte chemotaxis	2 of 45	2.64	0.0017
GO:0070098	chemokine-mediated signaling pathway	2 of 75	2.42	0.0017
GO:0044364	disruption of cells of other organism	2 of 89	2.34	0.0017
GO:0031640	killing of cells of other organism	2 of 89	2.34	0.0017
GO:0061844	antimicrobial humoral immune response mediated by antim...	2 of 107	2.26	0.0017

(more ...)

Molecular Function (Gene Ontology)

GO-term	description	count in network	strength	false discovery rate
GO:0008009	chemokine activity	2 of 48	2.61	0.00014

Cellular Component (Gene Ontology)

GO-term	description	count in network	strength	false discovery rate
GO:0005615	extracellular space	2 of 1134	1.24	0.0135

Reference publications (PubMed)

publication	(year) title	count in network	strength	false discovery rate
PMID:15212760	(2004) Lysophospholipids and chemokines activate distinct ...	2 of 3	3.81	0.00070
PMID:24273921	(2013) Expression of CC chemokines CCL2, CCL5, and CCL...	2 of 5	3.59	0.00074
PMID:19700933	(2009) Corneal and conjunctival fibroblasts are major sourc...	2 of 6	3.51	0.00074
PMID:18515987	(2008) The local cytokine and chemokine milieu within mali...	2 of 6	3.51	0.00074
PMID:16613763	(2006) Synthetic chemokines directly labeled with a fluores...	2 of 6	3.51	0.00074

(more ...)

local network cluster (STRING)

cluster	description	count in network	strength	false discovery rate
CL:7134	G alpha (i) signalling events	2 of 196	2.0	0.0016

KEGG Pathways

pathway	description	count in network	strength	false discovery rate
hsa04062	Chemokine signaling pathway	2 of 181	2.03	0.00043
hsa04060	Cytokine-cytokine receptor interaction	2 of 263	1.87	0.00046

Enriched GO functions for OSCC vs. control groups

explain columns

Biological Process (Gene Ontology)

GO-term	description	count in network	strength	false discovery rate
GO:0045073	regulation of chemokine biosynthetic process	2 of 11	2.55	0.00072
GO:0060444	branching involved in mammary gland duct morphogenesis	2 of 21	2.27	0.0014
GO:0010574	regulation of vascular endothelial growth factor production	2 of 32	2.09	0.0022
GO:0032722	positive regulation of chemokine production	3 of 56	2.02	0.00021
GO:0030574	collagen catabolic process	2 of 37	2.02	0.0026

(more ...)

Molecular Function (Gene Ontology)

GO-term	description	count in network	strength	false discovery rate
GO:0008009	chemokine activity	2 of 48	1.91	0.0023
GO:0005125	cytokine activity	5 of 216	1.66	2.16e-06
GO:0070851	growth factor receptor binding	3 of 131	1.65	0.00045
GO:0004222	metalloendopeptidase activity	2 of 110	1.55	0.0088
GO:0005126	cytokine receptor binding	4 of 272	1.46	0.00012

(more ...)

Cellular Component (Gene Ontology)

GO-term	description	count in network	strength	false discovery rate
GO:0005615	extracellular space	7 of 1134	1.08	2.01e-05
GO:0005576	extracellular region	8 of 2505	0.8	0.00011

Reference publications (PubMed)

publication	(year) title	count in network	strength	false discovery rate
PMID:27383261	(2016) Epithelial-mesenchymal interaction during photodyn...	3 of 3	3.29	4.70e-06
PMID:16520862	(2006) Sunscreens with an absorption maximum of > or =36...	3 of 3	3.29	4.70e-06
PMID:11876526	(2002) Direct role of human dermal fibroblasts and indirect ...	3 of 3	3.29	4.70e-06
PMID:9914427	(1999) Exposure of macrophage-like cells to titanium particl...	2 of 2	3.29	0.00015
PMID:9881751	(1998) Development of glycosylated human interleukin-1alp...	2 of 2	3.29	0.00015

(more ...)

local network cluster (STRING)

cluster	description	count in network	strength	false discovery rate
CL:914	Peptidase M10A, cysteine switch, zinc binding site	2 of 5	2.89	0.00028
CL:3634	interleukin-1-mediated signaling pathway, and TIR domain	2 of 60	1.81	0.0049
CL:7134	G alpha (i) signalling events	2 of 196	1.3	0.0187

KEGG Pathways

pathway	description	count in network	strength	false discovery rate
hsa05020	Prion diseases	2 of 33	2.07	0.0014
hsa05332	Graft-versus-host disease	2 of 36	2.04	0.0014
hsa05323	Rheumatoid arthritis	3 of 84	1.84	0.00039
hsa04657	IL-17 signaling pathway	3 of 92	1.8	0.00039
hsa05321	Inflammatory bowel disease (IBD)	2 of 62	1.8	0.0034

(more ...)

Enriched GO functions for OLP vs. OSCC groups

explain columns

Biological Process (Gene Ontology)

GO-term	description	count in network	strength	false discovery rate
GO:0010818	T cell chemotaxis	3 of 15	2.31	1.02e-05
GO:0072678	T cell migration	5 of 26	2.3	1.68e-08
GO:0048246	macrophage chemotaxis	2 of 14	2.17	0.00093
GO:0051770	positive regulation of nitric-oxide synthase biosynthetic pro...	2 of 16	2.11	0.0011
GO:0048247	lymphocyte chemotaxis	5 of 45	2.06	1.14e-07

(more ...)

Molecular Function (Gene Ontology)

GO-term	description	count in network	strength	false discovery rate
GO:0048248	CXCR3 chemokine receptor binding	3 of 5	2.79	5.14e-07
GO:0045236	CXCR chemokine receptor binding	4 of 16	2.41	4.01e-08
GO:0008009	chemokine activity	7 of 48	2.18	5.41e-12
GO:0005520	insulin-like growth factor binding	2 of 25	1.92	0.0019
GO:0048020	CCR chemokine receptor binding	3 of 41	1.88	9.91e-05

(more ...)

Cellular Component (Gene Ontology)

GO-term	description	count in network	strength	false discovery rate
GO:0009897	external side of plasma membrane	3 of 223	1.14	0.0487
GO:0005615	extracellular space	12 of 1134	1.04	2.98e-09
GO:0005576	extracellular region	16 of 2505	0.82	4.10e-10

Reference publications (PubMed)

publication	(year) title	count in network	strength	false discovery rate
PMID:27510253	(2016) Genetic polymorphisms of CCL2 associated with sus...	4 of 4	3.01	1.81e-08
PMID:25982097	(2015) The exaggerated inflammatory response in Behcet's ...	4 of 4	3.01	1.81e-08
PMID:21274880	(2011) Serum chemokine CXC ligand 10 (CXCL10) predicts f...	4 of 4	3.01	1.81e-08
PMID:17304353	(2007) Shear stress-induced changes in atherosclerotic pla...	4 of 4	3.01	1.81e-08
PMID:9730616	(1998) The human gene encoding SCYB9B, a putative novel ...	3 of 3	3.01	1.48e-06

(more ...)

local network cluster (STRING)

cluster	description	count in network	strength	false discovery rate
CL:7370	CXCR3 chemokine receptor binding	3 of 5	2.79	5.02e-07
CL:7361	Chemokine receptors bind chemokines	5 of 29	2.25	1.23e-08
CL:7364	CXC Chemokine domain, and dendritic cell chemotaxis	4 of 24	2.23	1.70e-07
CL:7488	CC chemokine, conserved site, and chemokine (C-C motif) li...	2 of 14	2.17	0.00071
CL:7134	G alpha (i) signalling events	7 of 196	1.57	1.87e-08

KEGG Pathways

pathway	description	count in network	strength	false discovery rate
hsa04668	TNF signaling pathway	5 of 108	1.68	1.11e-06
hsa04657	IL-17 signaling pathway	4 of 92	1.65	2.58e-05
hsa04620	Toll-like receptor signaling pathway	4 of 102	1.61	3.07e-05
hsa04062	Chemokine signaling pathway	7 of 181	1.6	7.98e-09
hsa04060	Cytokine-cytokine receptor interaction	10 of 263	1.59	1.01e-12

(more ...)

Enriched GO functions for OLK vs. OSCC groups

explain columns

Biological Process (Gene Ontology)

GO-term	description	count in network	strength	false discovery rate
GO:0001780	neutrophil homeostasis	2 of 11	2.65	0.0090
GO:0002828	regulation of type 2 immune response	2 of 30	2.21	0.0101
GO:0030574	collagen catabolic process	2 of 37	2.12	0.0101
GO:0031018	endocrine pancreas development	2 of 38	2.11	0.0101
GO:0002548	monocyte chemotaxis	2 of 41	2.08	0.0101

(more ...)

Molecular Function (Gene Ontology)

GO-term	description	count in network	strength	false discovery rate
GO:0004869	cysteine-type endopeptidase inhibitor activity	2 of 57	1.93	0.0200
GO:0004222	metalloendopeptidase activity	2 of 110	1.65	0.0200
GO:0004252	serine-type endopeptidase activity	2 of 180	1.43	0.0332
GO:0004857	enzyme inhibitor activity	3 of 388	1.28	0.0200
GO:0098772	molecular function regulator	5 of 1793	0.83	0.0200

Cellular Component (Gene Ontology)

GO-term	description	count in network	strength	false discovery rate
GO:0005615	extracellular space	5 of 1134	1.03	0.0025
GO:0005576	extracellular region	6 of 2505	0.77	0.0039

Reference publications (PubMed)

publication	(year) title	count in network	strength	false discovery rate
PMID:8486377	(1993) The order and orientation of a cluster of metalloprot...	2 of 2	3.39	0.0018
PMID:33243243	(2020) Multi-omics examination of Q fever fatigue syndrom...	2 of 2	3.39	0.0018
PMID:32725166	(2020) Salivary and Serum Inflammatory Profiles Reflect Dif...	2 of 2	3.39	0.0018
PMID:32698696	(2020) Mixture of enzyme-processed Panax ginseng and Ga...	2 of 2	3.39	0.0018
PMID:31734236	(2020) Intraluminal fluid infusion in a rat jejunum ischemiar...	2 of 2	3.39	0.0018

(more ...)

local network cluster (STRING)

cluster	description	count in network	strength	false discovery rate
CL:914	Peptidase M10A, cysteine switch, zinc binding site	2 of 5	2.99	0.00013
CL:32169	mixed, incl. Cadherin-like, and C2H2-type zinc finger	2 of 191	1.41	0.0187

KEGG Pathways

pathway	description	count in network	strength	false discovery rate
hsa01521	EGFR tyrosine kinase inhibitor resistance	2 of 78	1.8	0.0241
hsa05323	Rheumatoid arthritis	2 of 84	1.77	0.0241
hsa04657	IL-17 signaling pathway	2 of 92	1.73	0.0241
hsa04066	HIF-1 signaling pathway	2 of 98	1.7	0.0241
hsa04218	Cellular senescence	2 of 156	1.5	0.0241

Enriched GO functions for OLP vs. OLK groups

explain columns

Molecular Function (Gene Ontology)

GO-term	description	count in network	strength	false discovery rate
GO:0008083	growth factor activity	2 of 160	1.79	0.0033
GO:0008201	heparin binding	2 of 161	1.78	0.0033
GO:0048018	receptor ligand activity	3 of 458	1.51	0.0012
GO:0005102	signaling receptor binding	4 of 1513	1.11	0.0012

Cellular Component (Gene Ontology)

GO-term	description	count in network	strength	false discovery rate
GO:0005576	extracellular region	4 of 2505	0.89	0.0043

Reference publications (PubMed)

publication	(year) title	count in network	strength	false discovery rate
PMID:19339092	(2009) Kidney pathological changes in metabolic syndrome:...	2 of 2	3.69	0.0057
PMID:29284973	(2017) Successful Nonsurgical Management of Periapical L...	2 of 3	3.51	0.0057
PMID:19166774	(2009) An extract of green tea, epigallocatechin-3-gallate, re...	2 of 3	3.51	0.0057
PMID:18755182	(2008) The matrix protein CCN1 (CYR61) promotes prolifera...	2 of 3	3.51	0.0057
PMID:12386288	(2002) Midkine expression in the course of nephrogenesis a...	2 of 3	3.51	0.0057

(more ...)
