# Supplementary figures and images for: Examination of Oral Squamous Cell Carcinoma and Precancerous Lesions Using Proximity Extension Assay and Salivary RNA Quantification
Source: Biomedicines. 2020 Dec 14;8(12):610. doi: 10.3390/biomedicines8120610 (PMC7764999; doi:10.3390/biomedicines8120610)

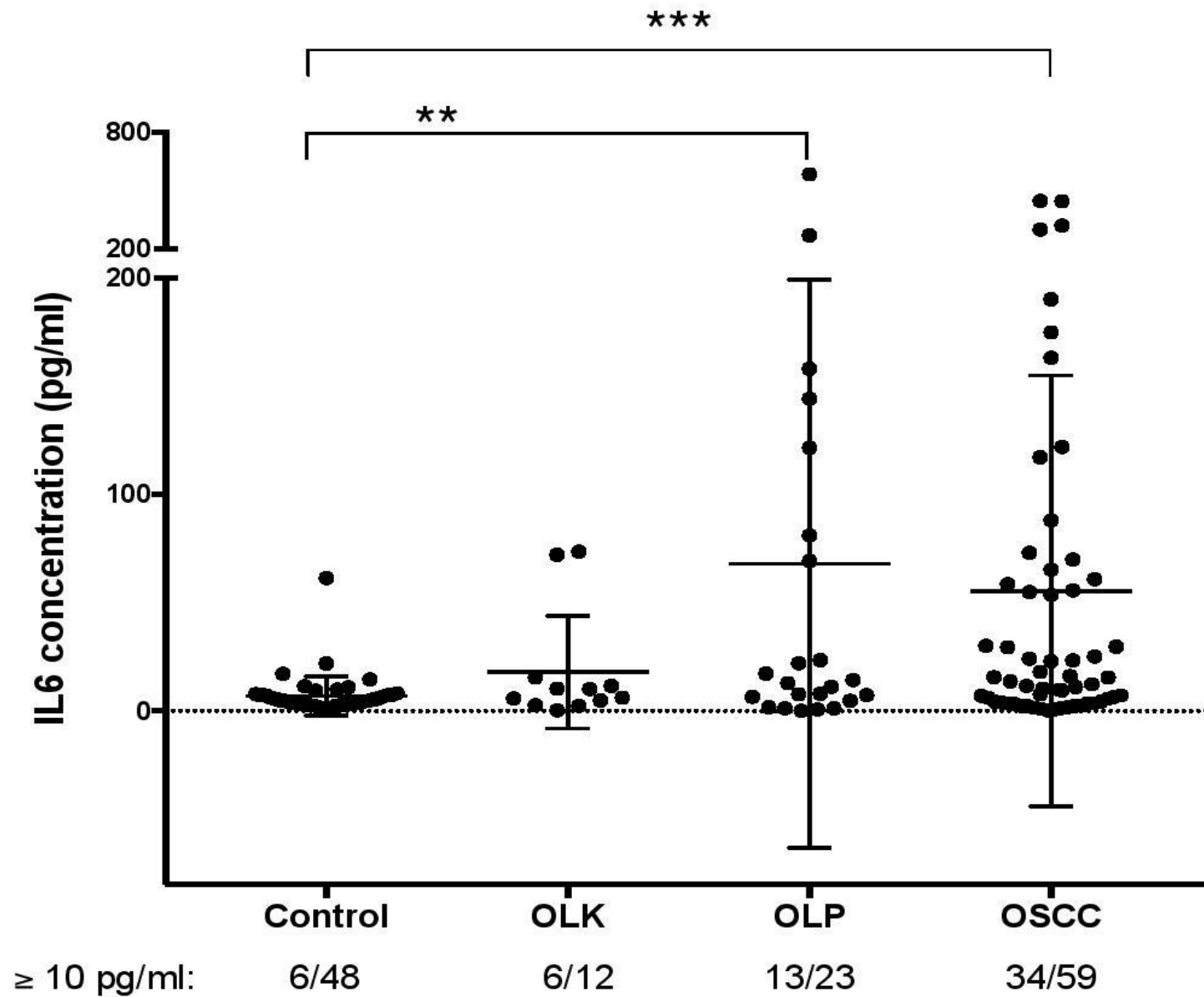

Supplement: Supplementary file 1 [file biomedicines-08-00610-s001.zip › FigureS3.pdf]
